# Tailoring the volatility and stability of oligopeptides

**DOI:** 10.1002/jms.3959

**Published:** 2017-07-24

**Authors:** J. Schätti, U. Sezer, S. Pedalino, J. P. Cotter, M. Arndt, M. Mayor, V. Köhler

**Affiliations:** ^1^ University of Basel Department of Chemistry Basel 4056 Switzerland; ^2^ University of Vienna Faculty of Physics Boltzmanngasse 5 1090 Vienna Austria; ^3^ Karlsruhe Institute of Technology Institute for Nanotechnology Hermann‐von‐Helmholtz‐Platz 1 76344 Eggenstein‐Leopoldshafen Germany

**Keywords:** fluorination, molecular beams, peptides, thermal evaporation, vuv ionization

## Abstract

Amino acids are essential building blocks of life, and fluorinated derivatives have gained interest in chemistry and medicine. Modern mass spectrometry has enabled the study of oligo‐ and polypeptides as isolated entities in the gas phase, but predominantly as singly or even multiply charged species. While laser desorption of neutral peptides into adiabatically expanding supersonic noble gas jets is possible, UV–VIS spectroscopy, electric or magnetic deflectometry as well as quantum interferometry would profit from the possibility to prepare thermally slow molecular beams. This has typically been precluded by the fragility of the peptide bond and the fact that a peptide would rather ‘fry’, i.e. denature and fragment than ‘fly’. Here, we explore how tailored perfluoroalkyl functionalization can reduce the intermolecular binding and thus increase the volatility of peptides and compare it to previously explored methylation, acylation and amidation of peptides. We show that this strategy is essential and enables the formation of thermal beams of intact neutral tripeptides, whereas only fragments were observed for an extensively fluoroalkyl‐decorated nonapeptide. © 2017 The Authors. *Journal of Mass Spectrometry* Published by John Wiley & Sons Ltd.

## Introduction

Since the early days of Otto Stern^1^ and Immanuel Estermann,^2^ neutral molecular beams have played a key role in fundamental studies of physics and physical chemistry.^3^, ^4^, ^5^ Experiments with isolated molecules in the gas phase have laid the ground for high‐precision spectroscopy,^6^, ^7^ molecule and cluster deflectometry^8^, ^9^, ^10^ and for an improved understanding of chemical reactions with quantum state control.^11^, ^12^ Modern molecular beam experiments have allowed setting new bounds on the electric dipole moment of the electron^13^, ^14^ and enabled the observation of quantum interference with clusters and molecules,^15^, ^16^ even with masses exceeding 10′000 amu.^17^ Modern research in molecular beam methods has recently focused on obtaining improved control over the motional and internal states of polyatomic molecules using selectors,^18^, ^19^ electrical,^20^, ^21^, ^22^, ^23^, ^24^ magnetic^25^ and mechanical^26^ decelerators as well as laser cooling of dimers and trimers.^27^, ^28^, ^29^ Polyatomic particles were even successfully trapped at mK temperatures.^22^, ^26^, ^30^, ^31^ Complementary to that, there has been a growing effort to prepare neutral beams of large molecules. Our present contribution addresses the question how to bring complex biomolecular building blocks into the gas phase.^32^ D. Gross and G. Grodsky reported on the sublimation and decomposition of unmodified amino acids and certain dipeptides in 1955.^33^, ^34^, ^35^, ^36^, ^37^, ^38^ Methylation and acylation of peptides have already been investigated in the late 60s and early 70s in combination with electron impact mass spectrometry (EI‐MS) as a means for increased volatility in sequence analysis of unknown proteins.^39^, ^40^, ^41^ Here, we aim at preparing neutral continuous beams of peptides at low velocity as required for spectroscopy, deflectometry and quantum interferometry or even nanostructuring using soft‐landing of individual biomolecules on surfaces. Even though one may argue that biomolecules are most naturally studied in an aqueous environment, it is meaningful to start from isolated species to which one may later add an increasing number of water molecules to compare their physical data with quantum chemical models.^42^, ^43^ Additionally, gas phase studies of biomolecules enable the elucidation of intrinsic folding preferences without interference of solvents or other molecules.^44^, ^45^, ^46^, ^47^

While some biomolecules or biomolecular moieties – such as nucleobases and some vitamins – can be readily sublimated or evaporated^48^ oligopeptides, proteins and oligonucleotides will rather fragment than fly when heated. To suppress fragmentation, one may reduce the heating time or add collisional cooling, once the molecules are airborne. Both ideas have been earlier explored for biomolecules injected into supersonic noble gas jets.^43^, ^49^, ^50^ This way, neutral intact macromolecules can be volatilized, but at the expense of being 300–1000 m/s fast, depending on the gas type and temperature. In contrast, velocities down to several 10 m/s have been achieved in buffer gas cooled sources^51^ for molecules up to stilbene or using laser‐induced acoustic desorption even for molecules beyond 10′000 amu.^52^, ^53^ However, the generation of thermally slow neutral beams of oligopeptides, which we take here as examples of relevant but fragile biomolecules, poses a considerable challenge.

Fluorination has gained increasing attention in medicinal chemistry over the last 50 years.^54^, ^55^ Around 20% of all pharmaceuticals contain at least one carbon–fluorine bond. Fluorine modification of single amino acids, peptides and proteins substantially alters their properties and provides new opportunities for peptide and protein design.^56^, ^57^ The strong electron‐withdrawing effect of fluorine lowers the *pk*_a_ value of proximal protons, affects hydrogen bonding and – despite the high polarization of the individual carbon fluorine bond – perfluoroalkyls exhibit a low overall dipole moment due to their inherent geometry. The very low polarizability of perfluoroalkyls results in very weak intermolecular dispersion forces and consequently low boiling points compared to hydrocarbons of similar mass.^58^

In our present work, we focus on the question how to derivatize biomolecular structures such that their sublimation enthalpy is reduced and their thermal stability increased. Perfluoroalkyl functionalization of individual molecules is expected to reduce the binding to neighbouring particles and surfaces because earlier studies have shown that it enhances the volatility of large organic compounds.^17^, ^59^, ^60^, ^61^ Here, we apply this strategy for the first time to oligopeptides and study its influence on their volatility.

## Flying rather than frying the peptide

**Figure 1:**
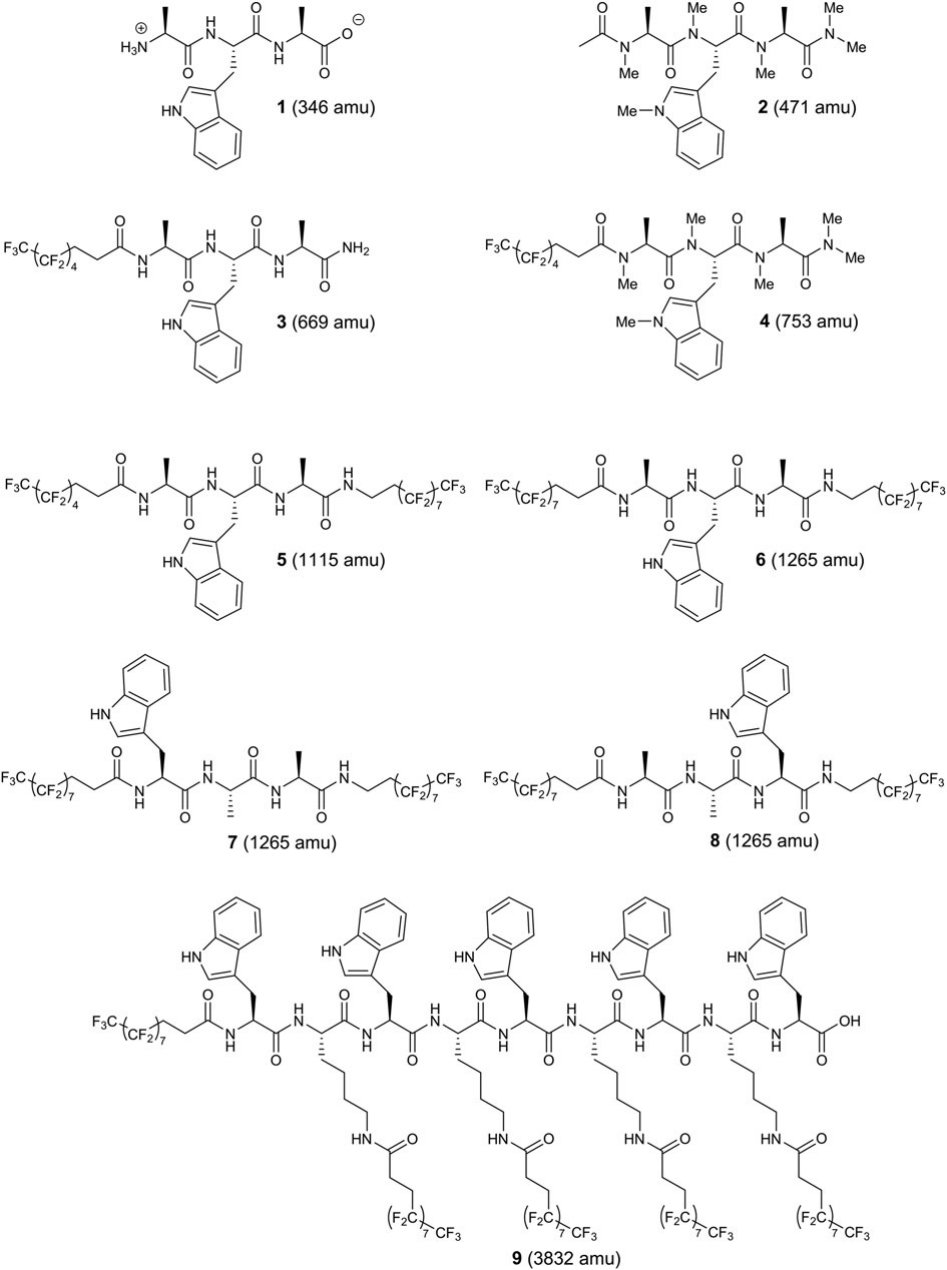
Gallery of peptides **1**–**9** with increasing molecular weight employed in this study. Tripeptides **1**–**8** resulted from variation of the Ala‐Trp‐Ala motif: charges and hydrogen bond donors present in parent tripeptide **1** were removed by acylation, methylation and amidation in derivative **2**; one perfluoroalkyl chain was introduced at the *N*‐terminus by acylation and the *C*‐terminus amidated in derivative **3**; **4** was obtained by methylation of **3**; fluorinated alkyl chains of different or equal length were introduced at the *N*‐ and *C*‐terminus by acylation and amidation, respectively, in derivative **5** and **6**; **7** and **8** are sequence isomers of **6**; high Trp and fluoroalkyl content realized by alternating Trp and Lys followed by acylation of the lysine side chains and the *N*‐terminus as exemplified in peptide **9**.

The volatility of the first model system, namely the tripeptide alanine–tryptophan–alanine (AWA) was compared in various modifications, i.e. in its native, methylated or perfluoroalkyl modified form including acylation and amidation of the termini, respectively, as shown in Fig. 1.

**Figure 2:**
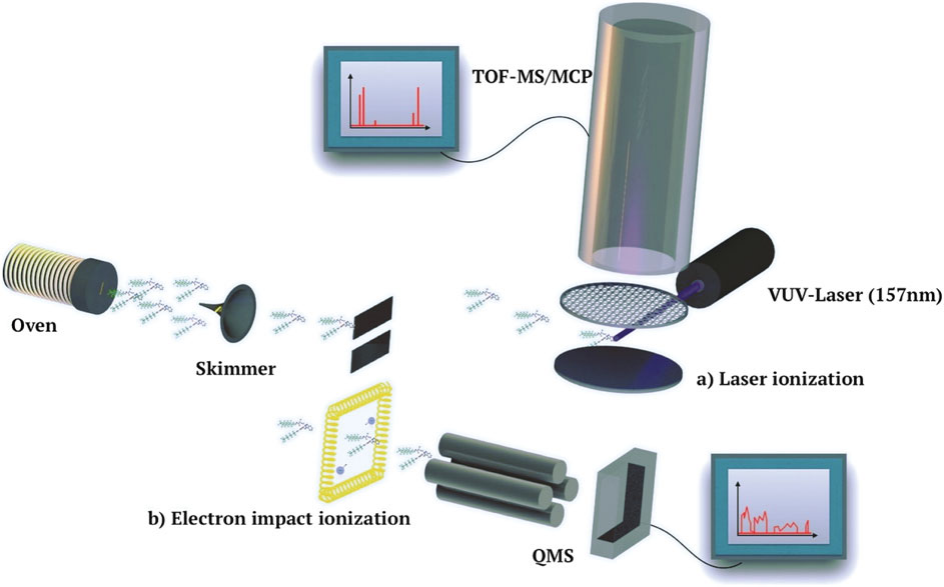
Experimental scheme for the volatilization/ionization tests. The peptides were heated in a ceramic cell with an aperture of 3 × 0.05 mm^**2**^. The molecular beam reached the mass spectrometer through two differential pumping stages, separated by one skimmer and one slit of 3 mm as the relevant dimension. Under heat load, the pressure in the three chambers was 1 **×** 10^−5^, 3 **×** 10^−6^ and 1.5 **×** 10^−7^ mbar, respectively. Pulsed photoionization of the molecular beam was combined with time‐of‐flight mass spectrometry (panel (a)). Alternatively, continuous electron impact ionization (b) was combined with a quadrupole mass spectrometer. Because both spectrometers were optimized for high transmission, their mass resolution is limited to about 2% with a calibration uncertainty of 5% across the entire mass range.

All peptides were volatilized in a resistively heated oven whose temperature was monitored on its outside and inside with an absolute uncertainty of ±5 K. The sublimated or evaporated molecules passed a differential pumping stage before they entered the probe chamber, where they were ionized (see Fig. 2). For selected compounds, electron impact ionization (*E*_impact_ = 70 eV) in quadrupole mass spectrometry (EI‐QMS) was compared with vacuum‐ultraviolet (VUV) photoionization (*λ* = 157 nm, *τ* = 10 ns) in time‐of‐flight mass spectrometry (TOF‐MS) to distinguish ionization induced from thermal decomposition processes.

## Results

**Figure 3:**
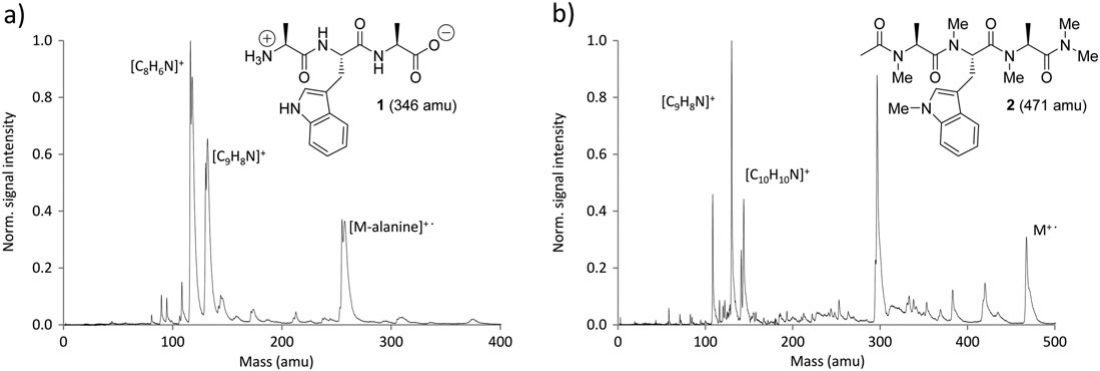
Panel (a) shows the mass spectrum of the native tripeptide Ala‐Trp‐Ala **1** after evaporation at 595 K and VUV postionization with a pulse intensity of *I*_ion_ = 2.9(3)MW/cm^2^. The native biomolecule (346 amu) falls apart under these conditions, and the following main fragments are observed: the indole cation (C_8_H_6_N^+^, 116 amu), the skatole cation (C_9_H_8_N^+^,130 amu) and a signal that is tentatively assigned to a cationic Ala‐Trp diketopiperazine fragment (C_14_H_15_N_3_O_2_^+^, 257 amu). The spectrum was calibrated to the indole cation (C_8_H_6_N^+^, 116 amu). (b) Under similar experimental conditions, but at lower temperature (*T* = 525 K), the mass spectrum of the methylated tripeptide 2 displays the intact parent ion at 471 amu. (b). Fragments include the *N*‐methyl indole cation (C_9_H_8_N^+^,130 amu) and the *N*‐methylated skatole cation (C_10_H_10_N^+^, 144 amu) as well as several unidentified species. The spectrum was calibrated to the *N*‐methyl indole cation (C_9_H_8_N^+^,130 amu).

We started by comparing the native tripeptide **1** with its methylated derivative **2** where internal charges were removed by acetylation and amidation of the termini (Fig. 3). Upon evaporation of **1** at varying source temperatures up to *T* = 595 K, only molecular fragments were detected, both in EI‐QMS and VUV‐TOF‐MS. Three major fragments were observed that were tentatively assigned to the loss of the C‐terminal alanine (H_2_N‐CH(CH_3_)‐COOH) possibly resulting from thermal diketopiperazine formation (C_14_H_15_N_3_O_2_, 257 amu)^62^, ^63^ as well as two common tryptophan fragments^64^ which were observed in all following mass spectra: the indole cation (C_8_H_6_N^+^, 116 amu) and the skatole cation (C_9_H_8_N^+^, 130 amu).

As reported in the literature, the removal of internal charges and hydrogen bond donors – through acylation of the *N*‐terminus, amidation of the *C*‐terminus and methylation of all nitrogen atoms – reduces the intermolecular binding and increases the volatility of the peptides.^39^ Indeed, evaporation of **2** permitted the observation of intact molecular ions (*m* = 471 amu), already at 525 K (see Fig. 3(b)).

Earlier studies with stable organic molecules showed that their volatility and stability can be enhanced by functionalization with perfluoroalkyl chains.^65^ The high electronegativity of fluorine reduces the polarizability‐to‐mass ratio in the compound and redistributes electron density slightly to the outside of the neutral molecule. Pictorially speaking, this functionalization aims at ‘wrapping’ the peptide in a protective fluorinated shell. Even though the particle is technically not encapsulated, the attachment of the chains is assumed to be beneficial.

**Figure 4:**
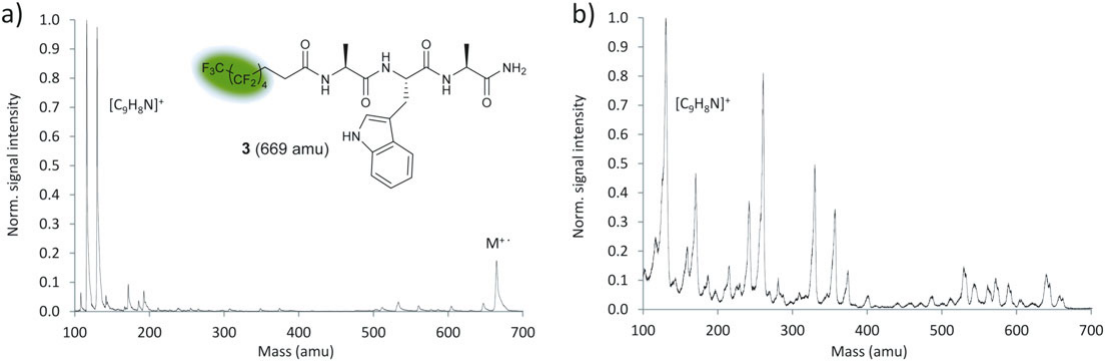
VUV‐TOF *versus* EI‐QMS. (a) VUV‐TOF mass spectrum of a thermal perfluoroalkyl functionalized peptide beam (**3**), recorded at *T* = 548 K. A strong parent peak (*m* = 669 amu) is observed and accompanied by the indole cation (C_8_H_6_N^+^, 116 amu) and skatole cation (C_9_H_8_N^+^, 130 amu). The spectrum was calibrated to the indole cation (C_8_H_6_N^+^, 116 amu). (b) In contrast, the EI‐QMS spectrum at 70‐eV electron energy yields a pronounced fragment spectrum under otherwise identical boundary conditions. The spectrum was calibrated to the skatole cation (C_9_H_8_N^+^, 130 amu). The green highlight indicates the fluoroalkyl‐tag. [Colour figure can be viewed at wileyonlinelibrary.com]

This hypothesis was verified for derivative **3** of the thermolabile tripeptide AWA. Upon heating to 548 K and exposure to VUV‐TOF mass spectrometry with laser settings equal to those used for the native peptide **1**, a substantial parent peak was observed (Fig. 4(a)) corroborating our design hypothesis. It was also noted that VUV photoionization is softer than electron impact ionization (Fig. 4(b)).^66^ This was apparent in the substantially reduced number of fragments and the higher parent‐to‐fragment ratio shown in Fig. 4(a).

**Figure 5:**
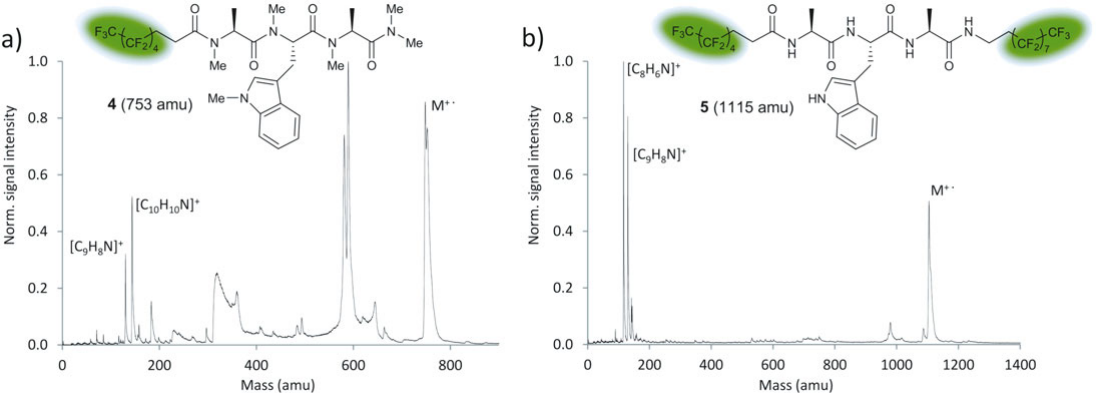
(a) VUV‐TOF mass spectrum of a thermal beam of perfluoroalkyl functionalized and methylated tripeptide **4** at *T* = 552 K and *I*_ion_ = 2.9(3) MW/cm^**2**^. We observe the methylated skatole cation (C_10_H_10_N^+^, 144 amu) and the *N*‐methyl indole cation (C_9_H_8_N^+^, 130 amu) as well as unidentified fragments. The spectrum was calibrated to the *N*‐methyl indole cation (C_9_H_8_N^+^,130 amu). (b) A second perfluoroalkyl chain adds to the molecular mass but results in a relatively clean mass spectrum of the non *N*‐methylated peptide **5** (compare also Fig. 4(a)). The spectrum was calibrated to the indole cation (C_8_H_6_N^+^, 116 amu). [Colour figure can be viewed at wileyonlinelibrary.com]

A dense mass spectrum was observed upon photoionization of a thermal beam of the methylated derivative **4**, which carries one fluoroalkyl chain introduced by acylation of the *N*‐terminus (Fig. 5(a)). Intact parent molecules were detected over the entire temperature range from 467 to 585 K. Based on this positive trend, additional perfluoroalkylation by amidation of the *C*‐terminus was investigated in the absence of *N*‐methylation. The mass spectrum of the peptide derivative **5** is shown in Fig. 5(b). The parent peak (*m* = 1115 amu) appears at 548 K, reaches its maximum at 586 K and now clearly dominates the spectrum. A similar but less pronounced effect is observed with the non‐*N*‐methylated peptide **3**, which carries only one fluoroalkyl chain (Fig. 4(a)). This corroborates the hypothesis that perfluoroalkylation enhances the volatility of the peptides and stabilizes them against thermal and photo‐induced dissociation whereas *N*‐methylation seems to promote fragmentation.

**Figure 6:**
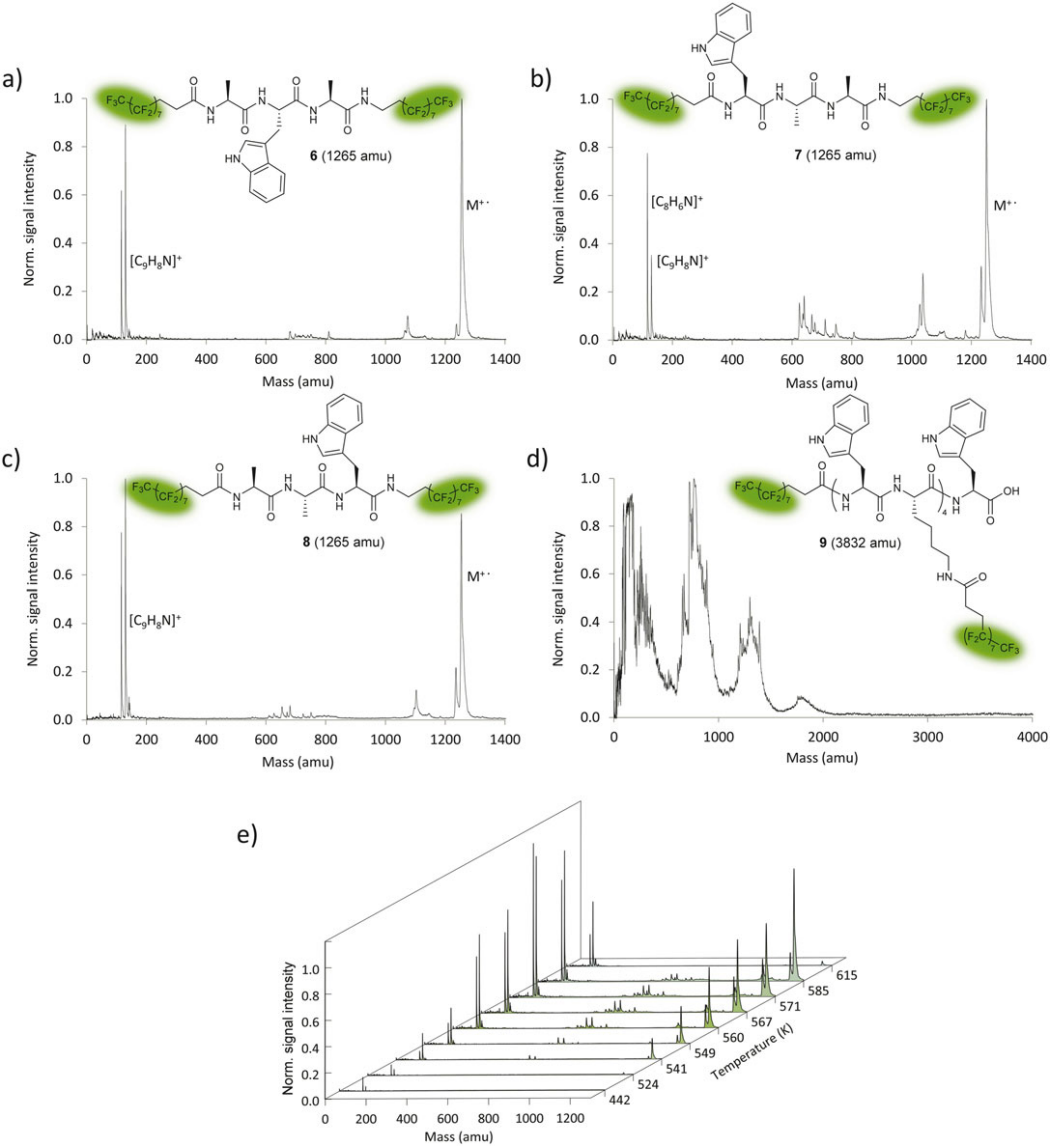
(a–c) Sequence isomers of tripeptide derivative with perfluoroalkyl decorated termini. Mass spectra for **6**, **7** and **8** are very similar, although a higher proportion of fragments is observed for **7**. Spectra were calibrated to the indole cation (C_8_H_6_N^+^, 116 amu). The indole cation is not indicated in (a) and (c) for improved clarity. (d) No intact ion was detected for highly perfluoroalkyl decorated Trp‐Lys oligomer **9** after thermal evaporation and photoionization. (e) Variation of the oven temperature leads to an increase of both the parent signal and the fragments of compound **8**. Significant thermal decomposition is observed at 615 K. [Colour figure can be viewed at wileyonlinelibrary.com]

To elucidate whether peptide stability and detectability are sequence dependent, the three perfluoroalkylated compounds **6**, **7** and **8** were synthesized, which differ only in the order of the three amino acid residues. Their VUV‐TOF mass spectra are shown in Fig. 6(a–c). The strongest parent signal is found in the temperature range of 575–585 K and for the highest photoionization intensity *I*_*ion*_ = 2.9(3)MW/cm^2^. In all three cases, we find a dominant parent peak (**1265** amu) and a simple mass pattern with the indole and skatole cation (C_8_H_6_N^+^, 116 amu and C_9_H_8_N^+^, 130 amu, respectively) as prominent fragments (Fig. 6(a–c)). Interestingly, the Trp‐Ala‐Ala sequence isomer **6** led to additional unidentified fragments over 600 amu compared to the other two sequence permutations **7** and **8** (Fig. 6(a)).

To probe the thermal contribution to fragmentation, we have studied the mass spectrum of **8** as a function of the source temperature (see Fig. 6(e)). Both the parent peak and its fragments rise in a constant relation with increasing temperature, up to 615 K, where the parent molecule finally disintegrates. This suggests that the molecules remain stable up to this temperature and that the fragments are predominantly due to the ionization process.

Because perfluoroalkyl functionalization substantially increases the molecular volatility,^65^ it is intriguing to test the mass limit of this method. The ideal model analyte would (i) have a high fluoroalkyl content and (ii) include a high tryptophan content for efficient photo‐ionization. Compound **9** fulfils these requirements and was heated in the same setup (Fig. 2), under high vacuum. The resulting VUV‐TOF‐mass spectrum (Fig. 6(d)) contains no intact parent molecule.

In order to test for the presence of an intact parent fraction in the neutral molecular beam, we have collected the evaporated material on a glass slide next to the oven. This sample was post‐analysed in a commercial MALDI instrument and did not show any intact parent peak, suggesting that the functionalized nonapeptide did not reach the glass slide as an intact entity in sufficient quantities.

## Discussion

By comparing the mass spectra of all tailored peptides depicted in Fig. 1 and assuming similar photoionization efficiency for compounds **1**–**8**, it can be seen that compounds **6**, **7** and **8** produce the most intense thermal beams and VUV‐TOF mass signals with a small proportion of fragments, even though they are 3.6 times more massive than the free AWA tripeptide alone. This observation can be attributed to reduced intermolecular interactions resulting from the removal of internal charges and the low polarizability of the perfluoroalkyl chains. The chain length of the perfluoroalkyl decoration, although not investigated in detail, seemed to play a minor but significant role with a more pronounced parent ion peak for compound **6** (C‐ and N‐terminal C_8_F_17_) in comparison to compound **5**, which carries a shorter perfluoroalkyl chain (C_5_F_11_) at the N‐terminus (Figs 5(b) and 6(a)). For compound **4** which has only one short perfluoroalkyl chain (C_5_F_11_), the relative intensity of the indole and skatole fragment ions *versus* the parental ion is significantly increased (Fig. 4(a)) although little additional fragments were observed. Interestingly, sequence isomers with identical decoration of perfluoroalkyl groups exhibited different degrees of fragmentation with the Trp‐Ala‐Ala sequence isomer being the most fragile in the series.

*N*‐methylation appears to promote fragmentation in combination with fluoroalkyl chains or without (Figs 3(b) and 5(a)). In contrast, already a single perfluoroalkyl chain with native N―H bonds in the peptide delivers significantly cleaner spectra than *N*‐methylated compounds.

The parent–fragment ratio does also depend on the ionization mechanism. Many mass spectrometry experiments use electron impact ionization with energies of 70 eV for optimum sensitivity. However, the excess of electron energy can open a variety of fragmentation channels.^67^ In contrast, VUV photoionization at 157 nm (7.90 eV) was confirmed as a soft ionization technique,^66^ whenever the peptide chain contained one or more tryptophan units.

Even though derivatization changes the geometry and chemical response of the molecule, we argue that our method can serve many practical purposes. In recent years, substantial research effort has been dedicated to evaluate, understand and explore the effects and benefits of fluorination in biochemistry, medicine and pharmacology.^54^, ^55^, ^56^, ^57^, ^68^ Studying fluoroalkyl derivatized molecules in the gas phase with an increasing number of hydration layers shall soon allow to systematically study the change of their electro‐optical properties in quantum interferometry experiments.^43^ Furthermore, it allows the preservation of native N―H functionality and thereby investigation of intramolecular interactions which might well reflect those of native peptides in the absence of solvent or other molecules.^44^, ^45^

## Methods

Synthesis and characterization of peptide constructs are detailed in the Supporting Information. Figures 3, 4, 5 and 6(a–d) were generated with Microsoft Excel 2010 using the ‘Scatter with Smooth Lines’ function. Figure 6e was prepared with Origin 9.2.214.

## Author contribution statement

J.S. and U.S. contributed equally to the work. J.S. synthesized and characterized the molecular compounds, U.S. and S.P designed and conducted the volatilization experiment with subsequent photoionization, J.C. designed and conducted the volatilization experiment with subsequent electron‐impact ionization, U.S., S.P. and J.C. analysed the data. V.K., M.A. and M. M. conceived and supervised the experiments. U.S., J.S., V.K. and M.A. wrote the paper, with all authors reviewing it.

## Additional information

There are no additional accession codes; the authors declare to have no competing financial interests.

## Supporting information


**Data S1.** Ala‐Trp‐Ala **1**. Top: 1H‐NMR (DMSO‐d6, 500 MHz, 293 K), Bottom: UV–Vis‐trace 190–500 nm of UPLC chromatogram (Method 1).
**S2.** Ac‐Ala‐Trp‐Ala‐NH2. Top: 1H‐NMR (DMSO‐d6, 500 MHz, 293 K), Bottom: UV–Vis‐trace 190–500 nm of UPLC chromatogram (Method 1).
**S3.** (2*H*,2*H*,3*H*,3*H*‐perfluorooctanoyl)‐Ala‐Trp‐Ala. Top: 1H‐NMR (DMSO‐d6, 500 MHz, 293 K), Bottom: UV–Vis‐trace 190–500 nm of UPLC chromatogram (Method 2).
**S4.** (2*H*,2*H*,3*H*,3*H*‐perfluoroundecanoyl)‐Ala‐Trp‐Ala. Top: 1H‐NMR (DMSO‐d6, 500 MHz, 293 K), Bottom: UV–Vistrace 190–500 nm of UPLC chromatogram (Method 2).
**S5.** (2*H*,2*H*,3*H*,3*H*‐perfluoroundecanoyl)‐Trp‐Ala‐Ala: Top: 1H‐NMR (DMSO‐d6, 500 MHz, 293 K), Bottom: UV‐Vistrace 190–500 nm of UPLC chromatogram (Method 2).
**S6.** (2*H*,2*H*,3*H*,3*H*‐perfluoroundecanoyl)‐Ala‐Ala‐Trp: Top: 1H‐NMR (DMSO‐d6, 500 MHz, 293 K), Bottom: UV‐Vistrace 190–500 nm of UPLC chromatogram (Method 2).
**S7.** Ac‐Nα‐Me‐Ala‐Nα‐Me‐Trp(Me)‐Nα‐Me‐Ala‐N(Me)2 **2**. Top: 1H–NMR (CDCl3, 500 MHz, 293 K), Bottom: UV–Vis‐trace 190–500 nm of UPLC chromatogram (Method 2).
**S8**. (2*H*,2*H*,3*H*,3*H*‐perfluorooctanoyl)‐Ala‐Trp‐Ala‐NH2. Top: 1H‐NMR (DMSO‐d6, 500 MHz, 293 K), Bottom: UV–Vistrace 190–500 nm of UPLC chromatogram (Method 2).
**S9**. (2*H*,2*H*,3*H*,3*H*‐perfluorooctanoyl)‐*Nα*‐Me‐Ala‐*Nα*‐Me‐Trp(Me)‐*Nα*‐Me‐Ala‐N(Me)2. Top: 1H‐NMR (CDCl3, 500 MHz, 293 K), Bottom: UV–Vis‐trace 190–500 nm of UPLC chromatogram (Method 2).
**S10.** (2*H*,2*H*,3*H*,3*H*‐perfluorooctanoyl)‐Ala‐Trp‐Ala‐(2*H*,2*H*,3*H*,3*H*‐perfluorodecylamid). Top: 1H‐NMR (DMSO‐d6, 500 MHz, 293 K), Bottom: UV–Vis‐trace 190–500 nm of UPLC chromatogram (Method 2).
**S11.** (2*H*,2*H*,3*H*,3*H*‐perfluoroundecanoyl)‐Ala‐Trp‐Ala‐(2*H*,2*H*,3*H*,3*H*‐perfluorodecylamid) **6**. Top: 1H‐NMR (DMF‐d7, 600 MHz, 333 K), Bottom: UV–Vis‐trace 190–500 nm of UPLC chromatogram (Method 2).
**S12**. (2*H*,2*H*,3*H*,3*H*‐perfluoroundecanoyl)‐Trp‐Ala‐Ala‐(2*H*,2*H*,3*H*,3*H*‐perfluorodecylamid) **7**. Top: 1H‐NMR (DMF‐d7, 600 MHz, 333 K), Bottom: UV–Vis‐trace 190–500 nm of UPLC chromatogram (Method 2).
**S13**. (2*H*,2*H*,3*H*,3*H*‐perfluoroundecanoyl) ‐Ala‐Ala‐Trp‐(2*H*,2*H*,3*H*,3*H*‐perfluorodecylamid) **8**. Top: 1H‐NMR (DMF‐d7, 600 MHz, 333 K), Bottom: UV–Vis‐trace 190–500 nm (UPLC, Method 2).
**S14**. *2H,2H,3H,3H*‐perfluorodecanoic acid NHS ester. 1H‐NMR (CDCl3, 500 MHz, 293 K).
**S15**. Trp‐Lys‐Trp‐Lys‐Trp‐Lys‐Trp‐Lys‐Trp. Top: 1H‐NMR (DMSO‐d6, 500 MHz, 293 K), Bottom: UV‐Vis‐trace 190–500 nm of UPLC chromatogram (Method 1). The chromatogram shows multiple peaks with identical absorption and MS‐spectra which are possibly caused by interconverting conformers.
**S16**. Compound **9**. Top: 1H‐NMR (HFIP‐d2+ 20 mol% H_2_O), presat (water and OH‐HFIP signal suppression, 600 MHz, 293 K), Bottom: 1H‐NMR (HFIP‐d2, 600 MHz, 293 K).
