# Supplementary material for: Tailoring the volatility and stability of oligopeptides
Source: J Mass Spectrom. 2017 Jul 24;52(8):550–6. doi: 10.1002/jms.3959 (PMC5601229; doi:10.1002/jms.3959)
Supplement: Supplementary file 1 — Data S1. Ala‐Trp‐Ala 1. Top: 1H‐NMR (DMSO‐d6, 500 MHz, 293 K), Bottom: UV–Vis‐trace 190–500 nm of UPLC chromatogram (Method 1). S2. Ac‐Ala‐Trp‐Ala‐NH2. Top: 1H‐NMR (DMSO‐d6, 500 MHz, 293 K), Bottom: UV–Vis‐trace 190–500 nm of UPLC chromatogram (Method 1). S3. (2H,2H,3H,3H‐perfluorooctanoyl)‐Ala‐Trp‐Ala. Top: 1H‐NMR (DMSO‐d6, 500 MHz, 293 K), Bottom: UV–Vis‐trace 190–500 nm of UPLC chromatogram (Method 2). S4. (2H,2H,3H,3H‐perfluoroundecanoyl)‐Ala‐Trp‐Ala. Top: 1H‐NMR (DMSO‐d6, 500 MHz, 293 K), Bottom: UV–Vistrace 190–500 nm of UPLC chromatogram (Method 2). S5. (2H,2H,3H,3H‐perfluoroundecanoyl)‐Trp‐Ala‐Ala: Top: 1H‐NMR (DMSO‐d6, 500 MHz, 293 K), Bottom: UV‐Vistrace 190–500 nm of UPLC chromatogram (Method 2). S6. (2H,2H,3H,3H‐perfluoroundecanoyl)‐Ala‐Ala‐Trp: Top: 1H‐NMR (DMSO‐d6, 500 MHz, 293 K), Bottom: UV‐Vistrace 190–500 nm of UPLC chromatogram (Method 2). S7. Ac‐Nα‐Me‐Ala‐Nα‐Me‐Trp(Me)‐Nα‐Me‐Ala‐N(Me)2 2. Top: 1H–NMR (CDCl3, 500 MHz, 293 K), Bottom: UV–Vis‐trace 190–500 nm of UPLC chromatogram (Method 2). S8. (2H,2H,3H,3H‐perfluorooctanoyl)‐Ala‐Trp‐Ala‐NH2. Top: 1H‐NMR (DMSO‐d6, 500 MHz, 293 K), Bottom: UV–Vistrace 190–500 nm of UPLC chromatogram (Method 2). S9. (2H,2H,3H,3H‐perfluorooctanoyl)‐Nα‐Me‐Ala‐Nα‐Me‐Trp(Me)‐Nα‐Me‐Ala‐N(Me)2. Top: 1H‐NMR (CDCl3, 500 MHz, 293 K), Bottom: UV–Vis‐trace 190–500 nm of UPLC chromatogram (Method 2). S10. (2H,2H,3H,3H‐perfluorooctanoyl)‐Ala‐Trp‐Ala‐(2H,2H,3H,3H‐perfluorodecylamid). Top: 1H‐NMR (DMSO‐d6, 500 MHz, 293 K), Bottom: UV–Vis‐trace 190–500 nm of UPLC chromatogram (Method 2). S11. (2H,2H,3H,3H‐perfluoroundecanoyl)‐Ala‐Trp‐Ala‐(2H,2H,3H,3H‐perfluorodecylamid) 6. Top: 1H‐NMR (DMF‐d7, 600 MHz, 333 K), Bottom: UV–Vis‐trace 190–500 nm of UPLC chromatogram (Method 2). S12. (2H,2H,3H,3H‐perfluoroundecanoyl)‐Trp‐Ala‐Ala‐(2H,2H,3H,3H‐perfluorodecylamid) 7. Top: 1H‐NMR (DMF‐d7, 600 MHz, 333 K), Bottom: UV–Vis‐trace 190–500 nm of UPLC chromatogram (Method 2). S13. (2H,2H,3H,3H‐perfluoroundecanoyl) ‐Ala‐Ala‐Trp‐(2H,2H,3H,3H‐perfluor [file JMS-52-550-s001.pdf]

Supplementary Information for:

Tailoring the volatility and stability of oligopeptides

Jonas Schätti[1], Ugur Sezer[2], Sebastian Pedalino[2], J. P. Cotter[2], Markus Arndt[2*], Marcel Mayor[1,3] and Valentin Köhler[1*]

[1]University of Basel, Department of Chemistry, Basel, 4056, Switzerland

[2]University of Vienna, Faculty of Physics, Vienna, 1090, Austria

[3]Karlsruhe Institute of Technology, Institute for Nanotechnology, Karlsruhe, 76021, Germany

*markus.arndt@univie.ac.at, valentin.koehler@unibas.ch

Table of Contents

Chemicals and instrumentation 3

General procedure 1: Ala-Trp-Ala **1**[1] 3

General procedure 2: Ac-Ala-Trp-Ala-NH_2 4

General procedure 3: Ac-N^{α}-Me-Ala-N^{α}-Me-Trp(Me)-N^{α}-Me-Ala-N$(Me)_2$ **2**[2] 6

(2*H*,2*H*,3*H*,3*H*-perfluorooctanoyl)-Ala-Trp-Ala-NH_2 **3** 7

(2*H*,2*H*,3*H*,3*H*-perfluorooctanoyl)-Ala-Trp-Ala 8

(2*H*,2*H*,3*H*,3*H*-perfluoroundecanoyl)-Ala-Trp-Ala 9

(2*H*,2*H*,3*H*,3*H*-perfluoroundecanoyl)-Trp-Ala-Ala 9

(2*H*,2*H*,3*H*,3*H*-perfluoroundecanoyl)-Ala-Ala-Trp 10

(2*H*,2*H*,3*H*,3*H*-perfluorooctanoyl)-N^{α}-Me-Ala-N^{α}-Me-Trp(Me)-N^{α}-Me-Ala-N$(Me)_2$ **4** 11

General procedure 4: (2*H*,2*H*,3*H*,3*H*-perfluorooctanoyl)-Ala-Trp-Ala-(2*H*,2*H*,3*H*,3*H*-perfluorodecylamid) **5** 12

(2*H*,2*H*,3*H*,3*H*-perfluoroundecanoyl)-Ala-Trp-Ala-(2*H*,2*H*,3*H*,3*H*-perfluorodecylamid) **6** 13

(2*H*,2*H*,3*H*,3*H*-perfluoroundecanoyl)-Trp-Ala-Ala-(2*H*,2*H*,3*H*,3*H*-perfluorodecylamid) **7** 13

(2*H*,2*H*,3*H*,3*H*-perfluoroundecanoyl)-Ala-Ala-Trp-(2*H*,2*H*,3*H*,3*H*-perfluorodecylamid) **8** 14

NHS ester of *2H,2H,3H,3H*-perfluorodecanoic acid[3] 15

Trp-Lys-Trp-Lys-Trp-Lys-Trp-Lys-Trp 15

Compound **9** 16

^{1}H NMR spectra and LC chromatograms 18

Ala-Trp-Ala **1** 18

Ac-Ala-Trp-Ala-NH_2 19
(2*H*,2*H*,3*H*,3*H*-perfluorooctanoyl)-Ala-Trp-Ala 20
(2*H*,2*H*,3*H*,3*H*-perfluoroundecanoyl)-Ala-Trp-Ala 21
(2*H*,2*H*,3*H*,3*H*-perfluoroundecanoyl)-Trp-Ala-Ala 22
(2*H*,2*H*,3*H*,3*H*-perfluoroundecanoyl)-Ala-Ala-Trp 23
Ac-N^{α}-Me-Ala-N^{α}-Me-Trp(Me)-N^{α}-Me-Ala-N$(Me)_2$ **2** 24
(2*H*,2*H*,3*H*,3*H*-perfluorooctanoyl)-Ala-Trp-Ala-NH_2 **3** 25
(2*H*,2*H*,3*H*,3*H*-perfluorooctanoyl)-N^{α}-Me-Ala-N^{α}-Me-Trp(Me)-N^{α}-Me-Ala-N$(Me)_2$ **4** 26
(2*H*,2*H*,3*H*,3*H*-perfluorooctanoyl)-Ala-Trp-Ala-(2*H*,2*H*,3*H*,3*H*-perfluorodecylamid) **5** 27
(2*H*,2*H*,3*H*,3*H*-perfluoroundecanoyl)-Ala-Trp-Ala-(2*H*,2*H*,3*H*,3*H*-perfluorodecylamid) **6** 28
(2*H*,2*H*,3*H*,3*H*-perfluoroundecanoyl)-Trp-Ala-Ala-(2*H*,2*H*,3*H*,3*H*-perfluorodecylamid) **7** 29
(2*H*,2*H*,3*H*,3*H*-perfluoroundecanoyl)-Ala-Ala-Trp-(2*H*,2*H*,3*H*,3*H*-perfluorodecylamid) **8** 30
NHS ester of *2H,2H,3H,3H*-perfluorodecanoic acid 31
Trp-Lys-Trp-Lys-Trp-Lys-Trp-Lys-Trp 32
Compound **9** 33
References 34

Chemicals and instrumentation

Chemicals were purchased from Sigma Aldrich, Fluorochem, Novabiochem, Bachem or P&M-Invest and used as received unless otherwise noted. UPLC experiments were performed with an Acquity UPLC H Class Bio from Waters equipped with a PDA and a SQ detector 2 with the following column: ACQUITY UPLC, HSS T3 1.8 µm, 2.1 x 100 mm. Solvents were water and acetonitrile, respectively, each containing 0.1 % formic acid, later on referred to as (A) and (B). The flow rate was set to 0.61 ml/min and the temperature to 40 °C. Method 1: 0 min – 100% A; 1 min – 100% A; 3 min – 80% A; 13.5 min – 20% A. Method 2: 0 min – 50% A; 1 min – 50% A; 7 min – 20% A; 10 min – 10% A. Mass detection was performed in scan mode for positive ions (Cone Voltage 40 V, Desolvation temperature: 600°C). A Water Prep LC 4000 System equipped with a Waters 2487: Dual λ Absorbance Detector as UV-Vis detector was used for preparative separations with the following column: Agilent: XDB-C18, 21.2 x 150 mm, 5 µm. Ultra pure water was prepared by means of an Ultra Ionic system from Milli-Q. ESI-HRMS experiments were conducted with Bruker maXis 4G. MALDI was recorded on a Bruker Daltonics Ultraflex II at the Molecular & Biomolecular Analysis Laboratory ETH Zürich. NMR experiments were performed at 20°C or 25°C on Bruker Avance III NMR spectrometers operating at 600, 500 or 400 MHz proton frequency. NMR spectrometers were equipped with direct (400 MHz) or inverse (600 MHz ,500 MHz) dual channel, broadband probe heads with *z* -gradients. ^{13}C and overlapping ^{1}H shifts were determined by 2D NMR experiments (COSY, HMBC and HMQC). ^{13}C shifts for CF_2 and CF_3 groups were not determined. TFA = trifluoroacetic acid; DIPEA = *N*,*N*-diisopropylethylamine; DMF = *N*,*N*-dimethylformamide; PyBOP = (Benzotriazol-1-yloxy)tripyrrolidinophosphonium hexafluorophosphate; NHS = *N*-hydroxysuccinimide; HSTU = *N*,*N*,*N'*,*N'*-tetramethyl-*O*-(*N*-succinimidyl)uronium hexafluorophosphate, HFIP = 1,1,1,3,3,3-hexafluoro-2-propanol.

General procedure 1: Ala-Trp-Ala **1**[1]

2-Chlorotrityl chloride resin (1 mmol/g, 700 mg) was placed in a plastic syringe equipped with a filter frit. The resin was washed with CH_2Cl_2 (~4 ml). Subsequently the syringe was filled with CH_2Cl_2 (~4 ml) and placed on a shaker for 15 min. The CH_2Cl_2 was exchanged and DIPEA (6.0 eq, 0.70 ml, 4.2 mmol), and Fmoc-ala-OH (2.00 eq., 436 mg, 1.40 mmol) were added. Note: equivalents refer to chlorotrityl groups on the resin (1 mmol/g). After the sample was agitated on a shaker for 1.5 h the reaction mixture was removed and the resin was washed with CH_2Cl_2 (~4 ml) and DMF (~4 ml). The unreacted binding sites on the resin were capped by adding MeOH-solution (~5 ml, 15% MeOH, 5% DIPEA, 80% CH_2Cl_2) followed by 15 min agitation. Subsequently the sample was washed with CH_2Cl_2 (~4 ml) and DMF (~4 ml). Next Fmoc deprotection solution (20 % piperidine in DMF) was added and shaking continued for 5 mins. This step was repeated three times. Note: In the following this procedure will be referred to as *Fmoc deprotection*. The sample was washed with DMF (~4 ml) and CH_2Cl_2 (~4 ml) and again with DMF (~4 ml). Between the different washing steps the sample was agitated on the shaker for ~30 sec. Note: In the following this procedure will be referred to as *washing.* After the *washing* DMF (~4 ml), DIPEA (6.0 eq, 0.70 ml, 4.2 mmol), Fmoc-Trp(Boc)-OH (2.00 eq. 737 mg, 1.40 mmol) and PyBOP (2.00 eq., 729 mg, 1.40 mmol) were

added. The mixture was agitated on a shaker for 2 h. Note: In the following this procedure will be referred to as *peptide coupling*. This was followed by *washing*, *Fmoc deprotection* and another *washing*. For the next peptide coupling DIPEA (6.0 eq., 0.70 ml, 4.2 mmol), Fmoc-Ala-OH (2.00 eq., 436 mg, 1.40 mmol) and PyBOP (2.00 eq., 729 mg, 1.40 mmol) were used. After *washing*, *Fmoc- deprotection* and another *washing* the resin was rinsed with CH_2Cl_2 (3 × ~4 ml) before the cleavage solution (5 ml, 96 % TFA, 3 % triisopropylsilane, 3 % H_2O) was added. For the cleavage procedure the mixture was agitated on the shaker for 1 h. Subsequently the cleavage solution was removed and the resin washed with TFA (1 ml). The TFA solution was concentrated on the rotary evaporator. Ice cold Et_2O (50 ml) was added and the formed precipitate collected by centrifugation (4400 rpm). Washing was repeated three times with Et_2O (20 ml) and the product obtained after drying as a white powder (195 mg, 0.564 mmol, 81 %).

UPLC-MS: Method 1, T_R = 3.61 min; m/z MS (ES+): 116.4 [50%], 258.4 [25%], 347.3 [100%, M + H^+], 369.2 [70 %, M + Na^+], 383.2 [25%, M + K^+].

ESI-HRMS: calculated for $C_{17}H_{23}N_4O_4^+$: 347.1714; found: 347.1719.

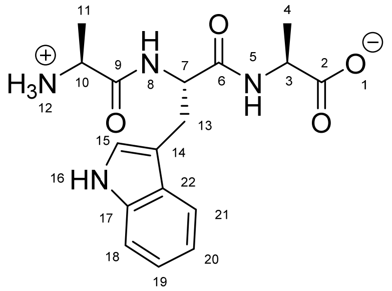

^{1}H-NMR (500 MHz, DMSO-d$_6$, 298 K): δ = 10.85 (d, $^3J_{H-H}$ = 2.2 Hz, 1 H, H-16), 8.52 (d, $^3J_{H-H}$ = 8.1 Hz, 1 H, H-8), 8.26 (d, $^3J_{H-H}$ = 7.0 Hz, 1 H, H-5), 7.66 (d, $^3J_{H-H}$ = 7.9 Hz, 1 H, H-21), 7.32 (dt, $^3J_{H-H}$ = 8.1 Hz, $^4J_{H-H}$ = 0.9 Hz, $^5J_{H-H}$ = 0.9 Hz, 1 H, H-18), 7.18 (d, $^3J_{H-H}$ = 2.2, 1 H, H-15), 7.06 (ddd, $^3J_{H-H}$ = 8.1 Hz, $^3J_{H-H}$ = 7.0Hz , $^4J_{H-H}$ = 1.2 Hz, 1 H, H-19), (ddd, $^3J_{H-H}$ = 8.0 Hz, $^3J_{H-H}$ = 7.0 Hz , $^4J_{H-H}$ = 0.9 Hz, 1 H, H-20), 4.59 (ddd , $^3J_{H-H}$ = 9.3 Hz, $^3J_{H-H}$ = 8.1 Hz, $^3J_{H-H}$ = 4.4 Hz, 1 H, H-7), 4.14 (p, $^3J_{H-H}$ = 7.1Hz, 1 H, H-3), 3.67 (q, $^3J_{H-H}$ = 6.9Hz, 1 H, H-10), 3.17 (dd, $^2J_{H-H}$ = 14.8 Hz, $^3J_{H-H}$ = 4.4 Hz, 1 H, H-13), 2.96 (dd, $^2J_{H-H}$ = 14.8 Hz, $^3J_{H-H}$ = 9.3 Hz, 1 H, H-13), 1.29 (d, $^3J_{H-H}$ = 7.0 Hz, 1 H, H-11), 1.27 (d, $^3J_{H-H}$ = 7.2 Hz, 1 H, H-4).

^{13}C δ = 174.1 (C-2), 171.1 (C-6), 170.9 (C-9), 136.6 (C-17), 127.6 (C-22), 123.7 (C-15), 120.6 (C-19), 118.3 (C-21), 118.0 (C-20), 111.1 (C-18), 110.1 (C-14), 53.1 (C-7), 48.1 (C-10), 47.7 (C-3), 27.5 (C-13), 17.3 (C-11), 17.2 (C-4).

General procedure 2: Ac-Ala-Trp-Ala-NH_2

Rink amid resin (0.54 mmol/g, 900 mg) was placed in a plastic syringe equipped with a filter frit. The resin was washed with CH_2Cl_2 (~4 ml). Then CH_2Cl_2 (~4 ml) was added and the syringe placed on a shaker for 15 min. Subsequently DMF (~4 ml), DIPEA (6.0 eq, 0.48 ml, 2.9 mmol), Fmoc-ala-OH (3.01 eq., 455 mg, 1.46 mmol) and PyBOP (3.00 eq., 760 mg, 1.46 mmol) were added. Note: Equivalents refer to the amine groups on the resin (0.54 mmol/g). The syringe was placed on a shaker and the mixture agitated

for 2 h. In the following this procedure will be referred to as *peptide coupling*. *Peptide coupling* was followed by *washing*, *Fmoc* deprotection and another *washing*. The second peptide coupling was performed with DIPEA (6.0 eq, 0.48 ml, 2.9 mmol), Fmoc-Trp(Boc)-OH (3.00 eq., 769 mg, 1.46 mmol) and PyBOP (3.00 eq., 760 mg, 1.46 mmol) followed by *washing*, *Fmoc deprotection* and another *washing*. The last peptide coupling was performed with DIPEA (6.0 eq, 0.48 ml, 2.9 mmol), Fmoc-Ala-OH (3.01 eq., 455 mg, 1.46 mmol) and PyBOP (3.00 eq., 760 mg, 1.46 mmol). After *washing*, *Fmoc deprotection* and another *washing*, capping solution (4 ml, 10 % acetic anhydride in DMF) and DIPEA (0.5 ml) were added and the mixture agitated for 5 min. The capping step was repeated once. After another *washing* the resin was rinsed with CH_2Cl_2 (3 × ~4 ml) before the cleavage solution (5 ml, 96 % TFA, 3 % triisopropylsilane, 3 % H_2O) was added. For the cleavage procedure the mixture was agitated on the shaker for 1 h. Subsequently the cleavage solution was collected and the resin washed with TFA (1 ml). The TFA solution was concentrated on the rotary evaporator. Ice cold Et_2O (50 ml) was added to the peptide solution and the precipitate was collected by centrifugation (4400 rpm). After washing with Et_2O (4 × 20 ml) and drying Ac-Ala-Trp-Ala-NH_2 was obtained as a white powder. (143 mg, 0.369 mmol, 76 %).

UPLC-MS: Method 1, T_R = 5.54 min; m/z MS (ES+): 371.3 [100%], 388.3 [40%, M + H^+], 410.4 [50 %, M + Na^+], 383.2 [25%, M + K^+].

ESI-HRMS: calculated for $C_{19}H_{26}N_5O_4^+$: 388.1979; found: 388.1979.

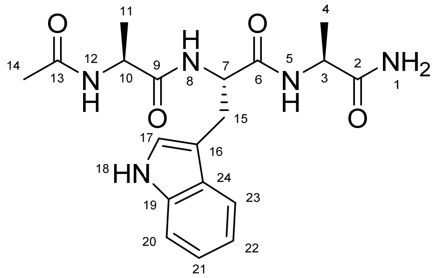

^{1}H-NMR (500 MHz, DMSO-d$_6$, 298 K): δ = 10.82 (d, $^3J_{H-H}$ = 2.3 Hz, 1 H, H-18), 8.04 (d, $^3J_{H-H}$ = 7.1 Hz, 1 H, H-12), 7.90 (d, $^3J_{H-H}$ = 7.7 Hz, 1 H, H-8), 7.80 (d, $^3J_{H-H}$ = 7.5 Hz, 1 H, H-5), 7.55 (dd, $^3J_{H-H}$ = 7.8 Hz, $^4J_{H-H}$ = 0.9 Hz, 1 H, H-23), 7.30 (dt, $^3J_{H-H}$ = 8.0, $^4J_{H-H}$ = 0.9 Hz, $^5J_{H-H}$ = 0.9 Hz, 1 H, H-20), 7.16 (s, 1 H, H-1), 7.14 (d, $^3J_{H-H}$ 2.4 Hz, 1 H, H-17), 7.05 (ddd, $^3J_{H-H}$ = 8.2 Hz, $^3J_{H-H}$ = 7.0 Hz, $^4J_{H-H}$ = 1.2 Hz, 1 H, H-21), 7.01 (s, 1 H, H-1), 6.96 (ddd, $^3J_{H-H}$ = 7.9 Hz, $^3J_{H-H}$ = 6.9 Hz, $^4J_{H-H}$ = 1.0 Hz, 1 H, H-22), 4.46 (td, $^3J_{H-H}$ = 8.4 Hz, $^3J_{H-H}$ = 4.6 Hz, 1 H, H-7), 4.19 (p, $^3J_{H-H}$ = 6.9 Hz, 1 H, H-10), 4.18 (p, $^3J_{H-H}$ = 7.2 Hz, 1 H, H-3), 3.15(dd, $^2J_{H-H}$ = 14.8 Hz, $^3J_{H-H}$ = 4.6 Hz, 1 H, H-15), 2.95 (dd, $^2J_{H-H}$ = 14.8 Hz, $^3J_{H-H}$ = 8.8 Hz, 1 H, H-15), 1.79 (s,3 H, H-14), 1.16 (d, $^3J_{H-H}$ = 7.1 Hz, 3 H, H-4), 1.13 (d, $^3J_{H-H}$ = 7.1 Hz, 3 H, H-11).

^{13}C δ = 173.8 (C-2), 172.3 (C-9), 170.6 (C-6), 196.2(C-13), 135.8 (C-19), 127.1 (C-24), 123.4 (C-17), 120.6 (C-22), 118.1 (C-23), 118.0 (C-21), 111.0 (C-20), 109.6 (C-16), 53.2 (C-7), 48.2 (C-10), 47.8 (C-3), 26.8 (C-15), 22.3 (C-14), 18.0 (C-4), 17.4 (C-11).

General procedure 3: Ac-N^{α}-Me-Ala-N^{α}-Me-Trp(Me)-N^{α}-Me-Ala-N(Me)$_2$ **2**[2]

NaH (25.0 eq., 465 mg, 19.4 mmol) was added to DMSO (14 mL). The suspension was heated to 90 °C for 60 min (until H_2 formation ceased) and allowed to cool to room temperature. The formed dimsyl-solution was added to a solution of Ac-Ala-Trp-Ala-NH$_2$ (1.00 eq., 300 mg, 0.774 mmol) in DMSO (5 ml). After 2 min methyl iodide (75.0 eq., 3.61 ml, 58.1 mmol) was added slowly under strong stirring. After 2 h H_2O (50 ml) was added to the reaction mixture and stirring continued for another 10 minutes. The product was extracted with chloroform (30 ml, 2 × 15 ml). The combined organic layers were washed with brine (2 × 25 ml) and H_2O (2 × 25 ml). The solvent was removed by means of a rotary evaporator and the crude product obtained as a brown oil. The product was purified by means of preparative HPLC using an isocratic flow of water (0.1 % TFA) /acetonitrile (0.1 % TFA) in a ratio of 75:25 with a flow rate of 19 ml (T_R = 11 min.). After lyophilisation Ac-N^{α}-Me-Ala-N^{α}-Me-Trp(Me)-N^{α}-Me-Ala-N(Me)$_2$ was isolated as a pale yellow solid (120 mg, 0.255 mmol, 33 %).

UPLC-MS: Method 1, T_R = 6.85 min; m/z MS (ES+): 342.3 [100%, M - (C-terminal Ala)$^-$], 427.4 [30%, M - NMe$_2^-$], 494.4 [50%, M + Na$^+$].

ESI-HRMS: calculated for $C_{25}H_{37}N_5NaO_4^+$: 494.2743; found: 494.2744.

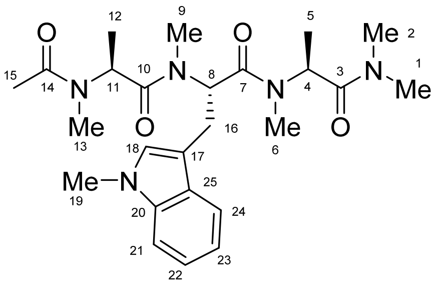

^{1}H-NMR (500 MHz, CDCl$_3$, 298 K): δ = 7.76 (dt, $^3J_{H-H}$ = 7.9 Hz, $^4J_{H-H}$ = 1.0, $^5J_{H-H}$ = 1.0 Hz, 1 H, H-24), 7.25 (dt, $^3J_{H-H}$ = 8.0 Hz, $^4J_{H-H}$ = 0.9 Hz, $^5J_{H-H}$ = 0.9 Hz, 1 H, H-21), 7.20 (ddd , $^3J_{H-H}$ = 8.1 Hz, $^3J_{H-H}$ = 6.9 Hz, $^4J_{H-H}$ = 1.2 Hz, 1 H, H-22), 7.11 (ddd , $^3J_{H-H}$ = 8.0 Hz, $^3J_{H-H}$ = 6.9 Hz, $^4J_{H-H}$ = 1.1 Hz, 1 H, H-23), 6.93 (s, 1 H, H-18), 5.88 (t, $^3J_{H-H}$ = 7.7, 1 H, H-8), 5.48 (q, $^3J_{H-H}$ = 6.9 Hz, 1 H, H-11), 5.40 (q, $^3J_{H-H}$ = 6.9 Hz, 1 H, H-4), 3.70 (s, 3 H, H-19), 3.30 (dd, $^2J_{H-H}$ = 14.5 Hz, $^3J_{H-H}$ = 7.7 Hz, 1 H, H-16), 3.09 (dd, $^2J_{H-H}$ = 14.5 Hz, $^3J_{H-H}$ = 8.1 Hz, 1 H, H-16), 2.98 (s, 3 H, H-9), 2.84 (s, 3 H, H-1 or H-2), 2.82(s, 3 H, H-6), 2.60 (s, 3 H, H-1 or H-2), 2.36 (s, 3 H, H-13), 1.90 (s, 3 H, H-15), 1.24 (d, $^3J_{H-H}$ = 6.8 Hz, 3 H, H-5), 1.20 (d, $^3J_{H-H}$= 6.8 Hz, 3 H, H-12).

^{13}C δ = 171.2 (C-10), 170.6 (C-3 and C-14), 196.5 (C-7), 136.7 (C-20), 128.0 (C-18 and C-25), 121.7 (C-22), 119.1 (C-23), 118.8 (C-24), 109.3 (C-17), 109.0 (C-21), 53.7 (C-8), 49.3 (C-4), 48.8 (C-11), 36.2 (C-1 or C-2), 35.8 (C-1 or C-2), 32.5 (C-19), 30.2 (C-9), 30.0 (C-13), 29.9 (C-6), 24.8 (C-16), 21.5 (C-15), 14.4 (C-5 and C-12).

(2*H*,2*H*,3*H*,3*H*-perfluorooctanoyl)-Ala-Trp-Ala-NH_2 **3**

The synthesis was performed in analogy to general procedure 2. Rink amid resin (900 mg) was placed in a plastic syringe equipped with a filter frit. The resin was washed with CH_2Cl_2 (~4 ml). Subsequently CH_2Cl_2 (~4 ml) was added and the mixture agitated on a shaker for 15 min. *Peptide coupling* was performed with Fmoc-Ala-OH (3.01 eq., 455 mg, 1.46 mmol) and the respective reagents as described for the Rink amid resin procedure above. Note: The equivalents refer to the amine groups on the resin (0.54 mmol/g). *Peptide coupling* was followed by *washing*, *Fmoc-deprotection* and another *washing*. The second *peptide coupling* was performed with Fmoc-Trp(Boc)-OH (3.00 eq., 769 mg, 1.46 mmol) and the respective reagents, followed by *washing*, *Fmoc-deprotection* and another *washing*. The final peptide coupling was performed Fmoc-Ala-OH (3.01 eq., 455 mg, 1.46 mmol) and the respective reagents and washing and deprotection steps. 2*H*,2*H*,3*H*,3*H*-perfluorooctanoic acid (3.00 eq., 499 mg, 1.46 mmol) was coupled with the peptide coupling procedure employing a reaction time of 4 hours. After *washing* the resin was rinsed with CH_2Cl_2 (3 × ~4 ml), cleavage performed and the acylated peptide obtained after precipitation and washing with Et_2O as described above. (235 mg, 0.338 mmol, 70 %).

UPLC-MS: Method 2, T_R = 1.89 min; m/z MS (ES+): 582.0 [10%], 653.1 [100%, M - NH_2^-], 670.0 [30%, M + H^+], 692.2 [80 %, M + Na^+].

ESI-HRMS: calculated for $C_{25}H_{27}F_{11}N_5O_4^+$: 670.1882; found: 670.1882.

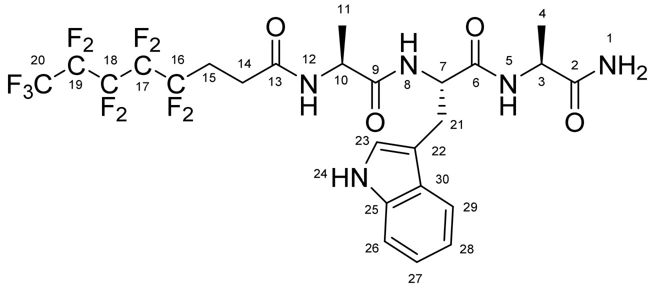

^{1}H-NMR (500 MHz, DMSO-d$_6$, 298 K): δ = 10.81 (d, $^3J_{H-H}$ = 2.4 Hz, 1 H, H-24), 8.26 (d, $^3J_{H-H}$ = 7.0 Hz, 1 H, H-12), 7.96 (d, $^3J_{H-H}$ = 7.7 Hz, 1 H, H-8), 7.79 (d, $^3J_{H-H}$ = 7.5 Hz, 1 H, H-5), 7.59 (dd, $^3J_{H-H}$ = 7.9 Hz, $^4J_{H-H}$ = 1.1 Hz, 1 H, H-29), 7.30 (dt, $^3J_{H-H}$ = 8.1, $^4J_{H-H}$ = 0.9 Hz, $^5J_{H-H}$ = 0.9 Hz, 1 H, H-26), 7.15 (s, 1 H, H-1), 7.13 (d, $^3J_{H-H}$ = 2.4 Hz, 1 H, H-23), 7.04 (ddd, $^3J_{H-H}$ = 8.2 Hz, $^3J_{H-H}$ = 7.0 Hz, $^4J_{H-H}$ = 1.2 Hz, 1 H, H-27), 7.02 (s, 1 H, H-1), 6.96 (ddd, $^3J_{H-H}$ = 8.0 Hz, $^3J_{H-H}$ = 7.0 Hz, $^4J_{H-H}$ = 1.0 Hz, 1 H, H-28), 4.47 (td, $^3J_{H-H}$ = 8.2, $^3J_{H-H}$ = 4.8 Hz, 1 H, H-7), 4.23 (p, $^3J_{H-H}$ = 6.9 Hz, 1 H, H-10), 4.18 (p, $^3J_{H-H}$ = 7.2 Hz, 1 H, H-3), 3.16 (dd, $^2J_{H-H}$ = 14.9, $^3J_{H-H}$ = 4.5 Hz, 1 H, H-21), 2.95 (dd, $^2J_{H-H}$ = 14.9 Hz, $^3J_{H-H}$ = 8.6 Hz, 1 H, H-21), 2.50-2.33 (m, 4 H, H-14 and H-15), 1.17 (d, $^3J_{H-H}$ = 7.2 Hz, 3 H, H-4), 1.16 (d, $^3J_{H-H}$ = 7.0 Hz, 3 H, H-11).

^{13}C δ = 174.4 (C-2), 172.0(C-9), 170.5 (C-6), 169.0 (C-13), 135.7 (C-25), 126.9(C-30), 123.5 (C-23), 120.7 (C-27), 118.3 (C-29), 118.1 (C-28), 111.2 (C-26), 109.5 (C-22), 53.3 (C-7), 48.4 (C-10), 47.9 (C-3), 26.9 (C-21), 25.6 (C-14), 25.6 (C-15), 17.9 (C-4), 17.6 (C-11).

(2*H*,2*H*,3*H*,3*H*-perfluorooctanoyl)-Ala-Trp-Ala

The synthesis was performed in analogy to general procedure 1 with the following quantities for the loading of the resin: 2-chlorotrityl chloride resin (700 mg), DIPEA (6.0 eq, 0.70 ml, 4.2 mmol), and Fmoc-ala-OH (2.00 eq., 436 mg, 1.40 mmol) were added. Note: The equivalents refer to the chlorotrityl groups on the resin. After the sample was agitated on a shaker for 1.5 h liquids were removed by filtration and the resin washed with CH_2Cl_2 (~4 ml) and DMF (~4 ml). The unreacted binding sites on the resin were capped by adding MeOH-solution (~5 ml, 15% MeOH, 5% DIPEA, 80% CH_2Cl_2) under agitation for 15 minutes. After *washing*, *Fmoc-deprotection* and another *washing, peptide coupling* was started with DIPEA (6.0 eq, 0.70 ml, 4.2 mmol), Fmoc-Trp(Boc)-OH (2.00 eq., 737 mg, 1.40 mmol) and PyBOP (2.00 eq., 729 mg, 1.40 mmol). This was followed by *washing*, *Fmoc-deprotection* and another *washing*. For the next *peptide coupling* the following quantities of DIPEA (6.0 eq, 0.70 ml, 4.2 mmol), Fmoc-ala-OH (2.00 eq., 436 mg, 1.40 mmol) and PyBOP (2.00 eq., 729 mg, 1.40 mmol) were used, followed by *washing*, *Fmoc-deprotection* and another *washing*. The last *peptide coupling* was performed with DIPEA (6.0 eq, 0.70 ml, 4.2 mmol) , 2*H*,2*H*,3*H*,3*H*-perfluorooctanoic acid (2.00 eq., 479 mg, 1.40 mmol), PyBOP (2.00 eq., 729 mg, 1.40 mmol) and a reaction time of 4 h. After *washing* the resin was washed with CH_2Cl_2 (3 × ~4 ml) before the cleavage solution (5ml, 96 % TFA, 3 % triisopropylsilane, 3 % H_2O) was added. Cleavage, precipitation and washing with Et_2O were performed as described in general procedure 1. The product was obtained as a white powder (310 mg, 0.462 mmol, 66%).

UPLC-MS: Method 1, T_R = 9.90 min; m/z MS (ES+): 582.2 [20%], 671.2 [100%, M + H^+], 693.2 [50%, M + Na^+], 709.0 [10 %, M + K^+].

ESI-HRMS: calculated for $C_{25}H_{26}F_{11}N_4O_5^+$: 671.1722; found: 671.1716.

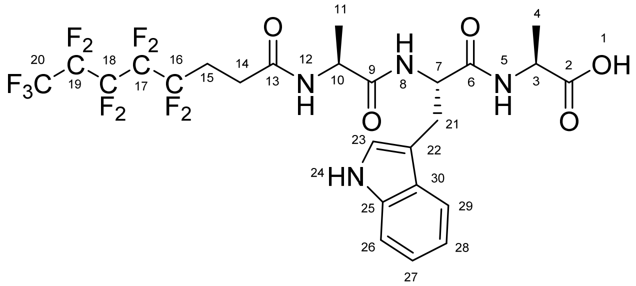

^{1}H-NMR (500 MHz, DMSO-d$_6$, 298 K): δ = 12.52 (s, 1 H, H-1), 10.80 (d, $^3J_{H-H}$ = 2.3 Hz, 1 H, H-24), 8.21 (d, $^3J_{H-H}$ = 7.2 Hz, 1 H, H-12), 8.11 (d, $^3J_{H-H}$ = 7.1 Hz, 1 H, H-5), 7.90 (d, $^3J_{H-H}$ = 8.2 Hz, 1 H, H-8), 7.59 (dd, $^3J_{H-H}$ = 8.0 Hz, $^4J_{H-H}$ = 1.2 Hz, 1 H, H-29), 7.30 (dt, $^3J_{H-H}$ = 8.0 Hz, $^4J_{H-H}$ = 0.9 Hz, $^5J_{H-H}$ = 0.9 Hz, 1 H, H-26), 7.13 (d, $^3J_{H-H}$ = 2.3 Hz, 1 H, H-23), 7.04 (ddd, $^3J_{H-H}$ = 8.1 Hz, J^3_{H-H} = 7.0 Hz, $^4J_{H-H}$ = 1.2 Hz, 1 H, H-27), 6.96 (ddd, $^3J_{H-H}$ = 8.0 Hz, $^3J_{H-H}$ = 7.0 Hz, $^4J_{H-H}$ = 1.0 Hz, 1 H, H-28), 4.53 (td, $^3J_{H-H}$ = 8.7 Hz, $^3J_{H-H}$ = 4.4 Hz, 1 H, H-7), 4.43 (p, $^3J_{H-H}$ = 7.1 Hz, 1 H, H-10), 4.41 (p, $^3J_{H-H}$ = 7.3 Hz, 1 H, H-3), 3.17 (dd, $^2J_{H-H}$ = 14.9 Hz, $^3J_{H-H}$ = 4.4 Hz, 1 H, H-21), 2.95 (dd, $^2J_{H-H}$ = 14.9 Hz, $^3J_{H-H}$ = 8.7 Hz, 1 H, H-21), 2.50-2.33 (m, 4 H, H-14 and H-15), 1.26 (d, $^3J_{H-H}$ = 7.3 Hz, 3 H, H-4), 1.13 (d, $^3J_{H-H}$ = 7.1 Hz, 3 H, H-11).

^{13}C δ = 173.7 (C-2), 171.6 (C-9), 170.6 (C-6), 169.0 (C-13), 135.7 (C-25), 127.1 (C-30), 123.5 (C-23), 120.6 (C-27), 118.3 (C-29), 118.0 (C-28), 111.1 (C-26), 109.7 (C-22), 52.8 (C-7), 48.2 (C-10), 47.3 (C-3), 27.1 (C-21), 25.5 (C-14), 25.5 (C-15), 17.7 (C-11), 16.9 (C-4).

(2*H*,2*H*,3*H*,3*H*-perfluoroundecanoyl)-Ala-Trp-Ala

Identical procedure as for (2*H*,2*H*,3*H*,3*H*-perfluorooctanoyl)-Ala-Trp-Ala but employing 2*H*,2*H*,3*H*,3*H*-perfluoroundecanoic acid (2.00 eq., 688 mg, 1.40 mmol) instead of 2*H*,2*H*,3*H*,3*H*-perfluorooctanoic acid. White powder. (395 mg, 0.482 mmol, 68.8%). The product contained possibly another compound or conformer (see Figure S4, Top).

UPLC-MS: Method 2, T_R = 4.30 min; m/z MS (ES+): 159.3 [20%], 276.2 [20%] 732.0 [20%], 821.1 [100%, M + H^+], 843.1 [80%, M + Na^+].

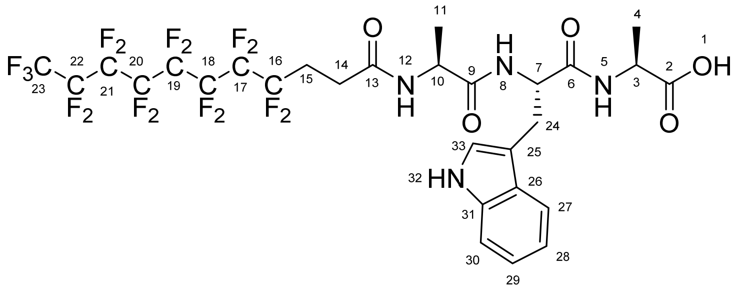

^{1}H-NMR (500 MHz, DMSO-d$_6$, 298 K): δ = 12.56 (s, 1 H, H-1), 10.80 (d, $^3J_{H-H}$= 2.4 Hz, 1 H, *H-32),* 8.20 (d, $^3J_{H-H}$ = 7.2 Hz, 1 H, H-12), 8.12 (d, $^3J_{H-H}$ = 7.2, Hz, 1 H, H-5), 7.88 (d, $^3J_{H-H}$ = 8.2 Hz, 1 H, H-8), 7.58 (dd, $^3J_{H-H}$ = 7.9 Hz, $^4J_{H-H}$= 1.0 Hz, 1 H, H-27), 7.30(dt, $^3J_{H-H}$ = 8.1 Hz, $^4J_{H-H}$= 1.0 Hz, $^5J_{H-H}$ = 1.0 Hz, 1 H, H-30), 7.13 (d, $^3J_{H-H}$ = 2.4 Hz, 1 H, H-33), 7.04 (ddd, $^3J_{H-H}$ = 8.1 Hz, 6.9 Hz, $^4J_{H-H}$= 1.0 Hz, 1 H, H-29), 6.95 (ddd, $^3J_{H-H}$ = 8.0 Hz, 6.9 Hz, $^4J_{H-H}$= 1.0 Hz, 1 H, H-28), 4.53 (td, $^3J_{H-H}$ = 8.7 Hz, 4.5 Hz, 1 H, H-7), 4.23 (p, $^3J_{H-H}$ = 7.2 Hz, 1 H, H-10), 4.21 (p, $^3J_{H-H}$ = 7.1 Hz, 1 H, H-3), 3.16 (dd, $^2J_{H-H}$ = 14.8 Hz, $^3J_{H-H}$ = 4.5 Hz, 1 H, H-24), 2.95 (dd, $^2J_{H-H}$ = 14.8 Hz, $^3J_{H-H}$ = 8.7 Hz, 1 H, H-24), 2.50-2.35 (m, 4 H, H-14 and H-15), 1.26 (d, $^3J_{H-H}$ = 7.2 Hz, 3 H, H-4), 1.13 (d, $^3J_{H-H}$ = 7.1 Hz, 3 H, H-11).

^{13}C δ = 173.6(C-2), 171.5 (C-9), 170.7 (C-6), 168.9 (C-13), 135.7 (C-31), 127.1(C-26), 123.4 (C-33), 120.6 (C-29), 118.3 (C-27), 117.9 (C-28), 111.0 (C-30), 109.6 (C-25), 52.8 (C-7), 47.5 (C-3 and C-10), 27.1 (C-24), 25.5 (C-14 and C-15), 17.7 (C-11), 16.9 (C-4).

(2*H*,2*H*,3*H*,3*H*-perfluoroundecanoyl)-Trp-Ala-Ala

Identical procedure as for (2*H*,2*H*,3*H*,3*H*-perfluoroundecanoyl)-Ala-Trp-Ala. White powder. (412 mg, 0.502 mmol, 72%).

UPLC-MS: Method 2, T_R = 4.46 min; m/z MS (ES+): 161.3 [20%], 731.9 [40%], 821.1 [100%, M + H^+], 843.0 [80%, M + Na^+].

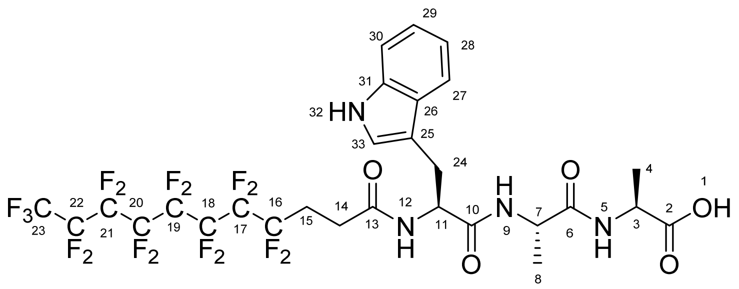

^{1}H-NMR (500 MHz, DMSO-d_6, 298 K): δ = 12.53 (s, 1 H, H-1), 10.79 (d, $^3J_{H-H}$= *2.4 Hz,* 1 H, H-32), 8.23 (d, $^3J_{H-H}$ = 8.1 Hz, 1 H, H-12), 8.11 (d, $^3J_{H-H}$ = 7.5, Hz, 1 H, H-9), 8.05 (d, $^3J_{H-H}$ = 7.2 Hz, 1 H, H-5), 7.60 (dd, $^3J_{H-H}$ = 7.9 Hz, $^4J_{H-H}$= 1.0 Hz, 1 H, H-27), 7.30(dd, $^3J_{H-H}$ = 8.1 Hz, $^4J_{H-H}$= 1.0 Hz, 1 H, H-30), 7.13 (d, $^3J_{H-H}$ = 2.4 Hz, 1 H, H-33), 7.04 (ddd, $^3J_{H-H}$ = 8.1 Hz, 7.0 Hz, $^4J_{H-H}$= 1.0 Hz, 1 H, H-29), 6.95 (ddd, $^3J_{H-H}$ = 7.9 Hz, 6.9 Hz, $^4J_{H-H}$= 1.0 Hz, 1 H, H-28), 4.56 (td, $^3J_{H-H}$ = 8.7 Hz, 4.6 Hz, 1 H, H-11), 4.31 (p, $^3J_{H-H}$ = 7.1 Hz, 1 H, H-7), 4.19 (p, $^3J_{H-H}$ = 7.3Hz, 1 H, H-3), 3.16 (dd, $^2J_{H-H}$ = 14.8 Hz, $^3J_{H-H}$ = 4.6 Hz, 1 H, H-24), 2.95 (dd, $^2J_{H-H}$ = 14.8 Hz, $^3J_{H-H}$ = 8.7 Hz, 1 H, H-24), 2.46-2.23 (m, 4 H, H-14 and H-15), 1.26 (d, $^3J_{H-H}$ = 7.3 Hz, 3 H, H-4), 1.20 (d, $^3J_{H-H}$ = 7.1 Hz, 3 H, H-8).

^{13}C δ = 173.8(C-2), 171.6 (C-6), 170.8 (C-10), 169.1 (C-13), 135.9 (C-31), 127.3(C-26), 123.5 (C-33), 120.6 (C-29), 118.3 (C-27), 117.9 (C-28), 111.0 (C-30), 109.9 (C-25), 53.4 (C-11), 47.6(C-7), 47.2 (C-3), 27.5 (C-24), 25.5 (C-14 and C-15), 17.8 (C-8), 16.8 (C-4).

(2*H*,2*H*,3*H*,3*H*-perfluoroundecanoyl)-Ala-Ala-Trp

Identical procedure as for (2*H*,2*H*,3*H*,3*H*-perfluoroundecanoyl)-Ala-Trp-Ala. White powder. (362 mg, 0.441 mmol, 63 %).

UPLC-MS: Method 2, T_R = 3.15 min; m/z MS (ES+): 205.2 [40%], 276.2 [20%], 821.1 [100%, M + H^+], 843.1 [70%, M + Na^+].

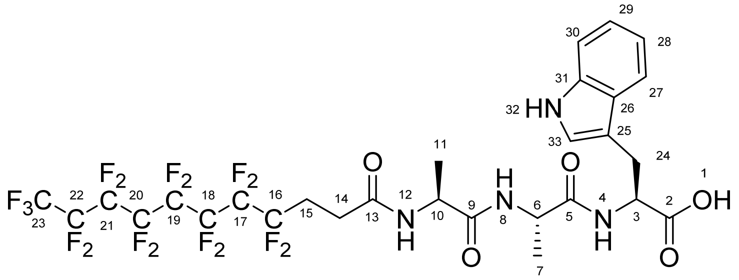

^{1}H-NMR (500 MHz, DMSO-d_6, 298 K): δ = 12.62 (s, 1 H, H-1), 10.84 (d, $^3J_{H-H}$= 2.4 Hz, 1 H, H-32), 8.23 (d, $^3J_{H-H}$ = 7.4 Hz, 1 H, H-12), 7.9 (d, $^3J_{H-H}$ = 7.7, Hz, 1 H, H-8), 7.97 (d, $^3J_{H-H}$ = 7.5 Hz, 1 H, H-4), 7.51 (dd, $^3J_{H-H}$ = 8.0

Hz, $^{4}J_{H-H}$= 1.0 Hz, 1 H, H-27), 7.32(dt, $^{3}J_{H-H}$ = 8.1 Hz, $^{4}J_{H-H}$= 1.0 Hz, $^{5}J_{H-H}$= 1.0 Hz, 1 H, H-30), 7.14 (d, $^{3}J_{H-H}$ = 2.4 Hz, 1 H, H-33), 7.05 (ddd, $^{3}J_{H-H}$ = 8.1 Hz, 6.9 Hz, $^{4}J_{H-H}$= 1.0 Hz, 1 H, H-29), 6.97 (ddd, $^{3}J_{H-H}$ = 8.0 Hz, 6.9 Hz, $^{4}J_{H-H}$= 1.0 Hz, 1 H, H-28), 4.60 (td, $^{3}J_{H-H}$ = 7.6 Hz, 5.4 Hz, 1 H, H-3), 4.30 (p, $^{3}J_{H-H}$ = 7.1 Hz, 1 H, H-6), 4.29 (p, $^{3}J_{H-H}$ = 7.0Hz, 1 H, H-10), 3.28 (dd, $^{2}J_{H-H}$ = 14.8 Hz, $^{3}J_{H-H}$ = 5.4 Hz, 1 H, H-24), 3.18 (dd, $^{2}J_{H-H}$ = 14.8 Hz, $^{3}J_{H-H}$ = 7.1 Hz, 1 H, H-24), 2.50-2.40 (m, 4 H, H-14 and H-15), 1.19 (d, $^{3}J_{H-H}$ = 7.1 Hz, 3 H, H-7), 1.15 (d, $^{3}J_{H-H}$ = 7.1 Hz, 3 H, H-11).

^{13}C δ = 173.5(C-2), 172.7 (C-5), 172.0 (C-9), 169.6 (C-13), 135.9 (C-31), 127.1(C-26), 123.4 (C-33), 120.7 (C-29), 118.1 (C-28), 117.9 (C-27), 111.1 (C-30), 109.4 (C-25), 52.6 (C-3), 47.8 (C-6 and C-10), 26.7 (C-24), 25.5 (C-14 and C-15), 17.9 (C-7), 17.8 (C-11).

(2*H*,2*H*,3*H*,3*H*-perfluorooctanoyl)-*N*$^{\alpha}$-Me-Ala-*N*$^{\alpha}$-Me-Trp(Me)-*N*$^{\alpha}$-Me-Ala-N(Me)$_2$ **4**

The dimsyl-anion was prepared in identical quantities and concentrations as described above for compound **2** and combined with a solution of (2*H*,2*H*,3*H*,3*H*-perfluorooctanoyl)-Ala-Trp-Ala-NH$_2$ (1.00 eq., 516 mg, 0.770 mmol) in DMSO (5 ml). Addition of MeI (75 eq., 3.6 ml, 58 mmol), quench with water, extraction and purification were performed as described above. The product was isolated as a pale brown oil (183 mg, 0.243 mmol, 31 %).

UPLC-MS: Method 2, T_R = 5.48 min; m/z MS (ES+): 624.3 [100%, M - (C-terminal Ala)$^-$], 709.3 [30%, M - NMe$_2^-$], 776.0 [80%, M + Na$^+$].

ESI-HRMS: calculated for $C_{31}H_{38}F_{11}N_5NaO_4^+$: 776.2640; found: 776.2651.

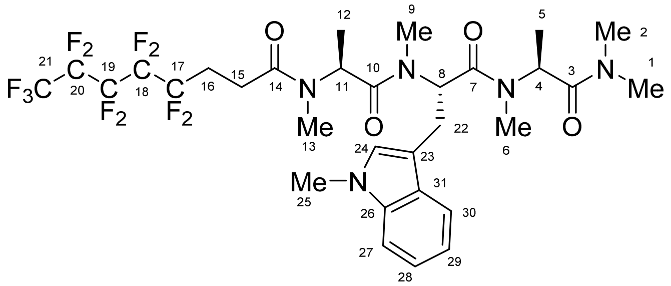

^{1}H-NMR (500 MHz, CDCl$_3$, 298 K): δ = 7.64 (dt, $^{3}J_{H-H}$ = 8.0 Hz, $^{4}J_{H-H}$ = 1.0 Hz, J^{5}_{H-H} = 1.0 Hz, 1 H, H-30), 7.26 (dt, $^{3}J_{H-H}$ = 8.3 Hz, $^{4}J_{H-H}$ = 1.0 Hz, $^{5}J_{H-H}$ = 1.0 Hz 1 H, H-27), 7.20 (ddd , $^{3}J_{H-H}$ = 8.1 Hz, $^{3}J_{H-H}$ = 6.9 Hz, $^{4}J_{H-H}$ = 1.0 Hz, 1 H, H-28), 7.10 (ddd , $^{3}J_{H-H}$ = 8.0 Hz, $^{3}J_{H-H}$ = 6.9 Hz, $^{4}J_{H-H}$ = 1.0 Hz, 1 H, H-29), 6.93 (s, 1 H, H-24), 5.88 (dd, $^{3}J_{H-H}$ = 7.2 Hz, $^{3}J_{H-H}$ = 8.5 Hz, 1 H, H-8), 5.46 (q, $^{3}J_{H-H}$ = 6.9 Hz, 1 H, H-11), 5.41 (q, $^{3}J_{H-H}$ = 6.9 Hz, 1 H, H-4), 3.70 (s, 3 H, H-25), 3.26 (dd, $^{2}J_{H-H}$ = 14.7 Hz, $^{2}J_{H-H}$ = 7.3 Hz, 1 H, H-22), 3.13 (dd, $^{2}J_{H-H}$ = 14.7 Hz, $^{3}J_{H-H}$ = 8.4 Hz, 1 H, H-22), 2.98 (s, 3 H, H-9), 2.86 (s, 6 H, (H-1 or H-2) and H-6), 2.65 (s, 3 H, H-1 or H-2), 2.52-2.40 (m, 2 H, H-16), 2.40-2.20 (m, 2 H, H-15), 2.30 (s, 3 H, H-13), 1.25 (d, $^{3}J_{H-H}$ = 6.9 Hz, 3 H, H-5), 1.19 (d, $^{3}J_{H-H}$ = 6.9 Hz, 3H, H-12).

^{13}C δ = 171.1(C-10), 170.7 (C-3), 169.7 (C-7), 169.5 (C-14), 136.7 (C-26), 128.0 (C-31), 127.9 (C-24), 121.7 (C-28), 119.1 (C-29), 118.7 (C-30), 109.3 (C-23), 109.2 (C-27), 53.9 (C-8), 49.2 (C-4), 49.1 (C-11), 36.3 (C-1 or C-2), 35.7 (C-1 or C-2), 32.4 (C-25), 30.2 (C-9), 29.9 (C-6), 29.0 (C-13), 26.4 (C-16), 24.6 (C-22), 24.2 (C-15), 14.4 (C-5 and C-12).

General procedure 4: (2*H*,2*H*,3*H*,3*H*-perfluorooctanoyl)-Ala-Trp-Ala-(2*H*,2*H*,3*H*,3*H*-perfluorodecylamid) **5**

(2*H*,2*H*,3*H*,3*H*-perfluorooctanoyl)-Ala-Trp-Ala (1.00 eq., 2.50 g, 3.73 mmol), PyBOP (1.50 eq., 2.91 g, 5.59 mmol) and 1*H*,1*H*,2*H*,2*H*-heptadecafluorodecylamine (1.50 eq., 2.59 g, 5.59 mmol) were dissolved in DMF (250 ml) and stirred for 3 h. Then H_2O (250 ml) was added and the precipitate collected by centrifugation (4400 rpm). The precipitate was washed with DMF (2 × 10 ml), H_2O (2 × 30 ml) and MeCN (4 × 30 ml). The crude product was dissolved in hot DMF (75 °C) and then reprecipitated by addition of ice cold water. After washing with H_2O (2 × 30 ml) and MeCN (4 × 30 ml) followed by drying (2*H*,2*H*,3*H*,3*H*-perfluorooctanoyl)-Ala-Trp-Ala-(2*H*,2*H*,3*H*,3*H*-perfluorodecylamin) was isolated as a white powder (1.82 g, 1.63 mmol, 44 %).

UPLC-MS: Method 2, T_R = 8.05 min; m/z MS (ES+): 653.1 [30%], 1116.1 [40%, M + H^+], 1138.0 [100%, M + Na^+], 1154.1 [10%, M + K^+].

ESI-HRMS: calculated for $C_{35}H_{30}F_{28}N_5O_4^+$: 1116.1845; found: 1116.1848.

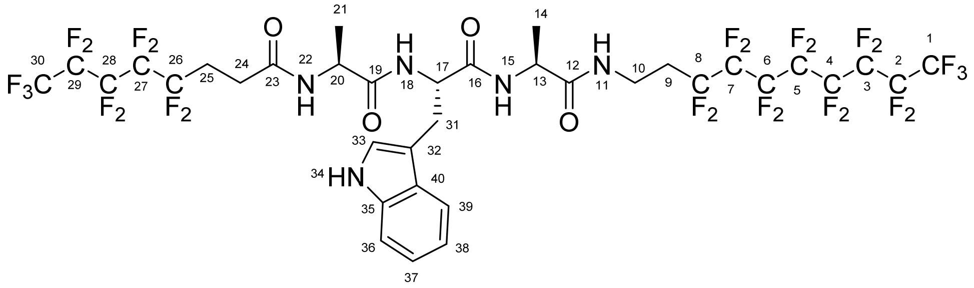

^{1}H-NMR (500 MHz, DMSO-d$_6$, 298 K): δ = 10.75 (s, 1H, H-34), 8.25 (d, $^3J_{H-H}$ = 6.6 Hz, 1 H, H-22), 7.89 (d, $^3J_{H-H}$ = 7.7 Hz, 1 H, H-18), 7.82 (d, $^3J_{H-H}$ = 7.2 Hz, 1 H, H-15), 7.76 (t, $^3J_{H-H}$ = 5.8 Hz, 1 H, H-11), 7.53 (d, $^3J_{H-H}$ = 7.9 Hz, 1 H, H-39), 7.28 (d, $^3J_{H-H}$ = 8.1 Hz, 1 H, H-36), 7.11 (s, 1 H, H-33), 7.01 (t, $^3J_{H-H}$ = 7.5 Hz, 1 H, H-37), 6.93 (t, $^3J_{H-H}$ = 7.5 Hz, 1 H, H-38), 4.48 (ddd, $^3J_{H-H}$ = 8.6 Hz, $^3J_{H-H}$ = 7.7 Hz , $^3J_{H-H}$ = 5.0 Hz, 1 H, H-17), 4.21-4.11 (m, 2 H, H-20 and H-13), 3.38-3.26 (m, 2 H, H-10), 3.16 (dd, $^2J_{H-H}$ = 14.9, Hz, $^3J_{H-H}$ = 5.0 Hz, 1 H, H-31), 3.00 (dd, $^2J_{H-H}$ = 14.9, Hz, $^3J_{H-H}$ = 8.6 Hz, 1 H, H-31), 2.50-2.23 (m, 6 H, H-9, H-24, H-25), 1.13 (d, $^3J_{H-H}$ = 7.1 Hz, 6 H, H-21, H-14).

^{13}C δ = 173.2 (C-19), 172.9 (C-12), 171.7 (C-16), 170.4 (C-23), 136.8 (C-35), 128.0 (C-40), 124.0 (C-33), 121.2 (C-37), 118.8 (C-39), 118.7 (C-38), 111.7 (C-36), 110.6 (C-32), 54.0 (C-17), 49.5 (C-20), 48.8 (C-13), 31.4 (C-10), 30.0 (C-9), 27.3 (C-31), 26.1 (C-24), 26.1 (C-25), 17.7 (C-14,C-21).

(2*H*,2*H*,3*H*,3*H*-perfluoroundecanoyl)-Ala-Trp-Ala-(2*H*,2*H*,3*H*,3*H*-perfluorodecylamid) **6**

Identical procedure as for (2H,2H,3H,3H-perfluorooctanoyl)-Ala-Trp-Ala-(2H,2H,3H,3H-perfluorodecylamid) but employing (2*H*,2*H*,3*H*,3*H*-perfluoroundecanoyl)-Ala-Trp-Ala instead of (2*H*,2*H*,3*H*,3*H*-perfluorooctanoyl)-Ala-Trp-Ala. White powder (710 mg, 0.561 mmol, 46 %).

UPLC-MS: Method 2, T_R = 9.65 min; m/z MS (ES+): 803.0 [20%], 1265.9 [20%, M + H^+], 1288.0 [100%, M + Na^+], 1303.8 [10%, M + K^+].

ESI-HRMS: calculated for $C_{38}H_{30}F_{34}N_5O_4^+$: 1266.1749; found: 1266.1739.

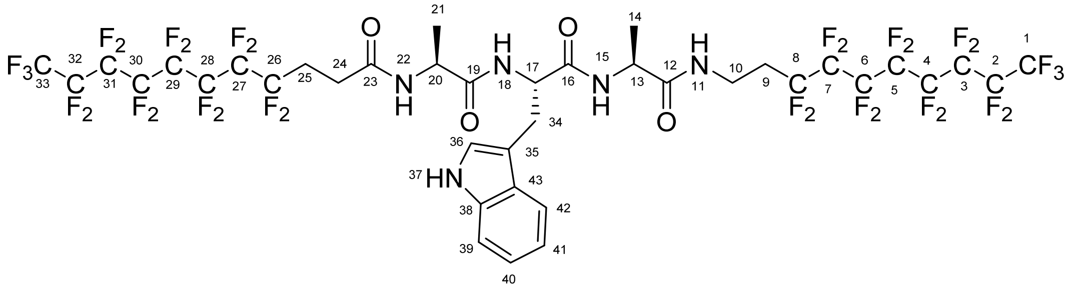

^{1}H-NMR (600 MHz, DMF-d_7, 333 K): δ = 10.73 (s, 1 H, H-37), 8.20 (d, $^3J_{H-H}$ = 6.2 Hz, 1 H, H-22), 7.82 (d, $^3J_{H-H}$ = 7.3 Hz, 1 H, H-18), 7.63 (d, $^3J_{H-H}$ = 7.5 Hz, 1 H, H-15), 7.60 (d, $^3J_{H-H}$ = 8.0 Hz, 1 H, H-42), 7.58 (t, $^3J_{H-H}$ = 5.7 Hz, 1 H, H-11), 7.39 (d, $^3J_{H-H}$ = 8.1 Hz, 1 H, H-39), 7.24 (d, $^3J_{H-H}$ = 2.3 Hz, 1 H, H-36), 7.08 (ddd, $^3J_{H-H}$ = 8.0 Hz, $^3J_{H-H}$ = 7.2 Hz, $^4J_{H-H}$ = 1.2 Hz, 1 H, H-40), 7.01 (ddd, $^3J_{H-H}$ = 8.0 Hz $^3J_{H-H}$ = 7.0 Hz, $^4J_{H-H}$ = 1.0 Hz, 1 H, H-41), 4.60 (td, $^3J_{H-H}$ = 7.6 Hz, $^3J_{H-H}$ = 5.3 Hz, 1 H, H-17), 4.33 (p, $^3J_{H-H}$ = 7.1 Hz, 1 H, H-13), 4.30 (p, $^3J_{H-H}$ = 7.0 Hz, 1 H, H-20), 3.54-3.43 (m, 2 H, H-10), 3.30 (dd, $^2J_{H-H}$ = 14.8 Hz, $^3J_{H-H}$ = 5.2 Hz, 1 H, H-34), 3.18 (dd, $^2J_{H-H}$ = 14.8 Hz, $^3J_{H-H}$ = 8.1 Hz, 1 H, H-34), 2.63-2.43 (m, 6 H, H-24, H-25 and H-9), 1.29 (d, $^3J_{H-H}$ = 7.1 Hz, 3 H, H-21), 1.24 (d, $^3J_{H-H}$ = 7.1 Hz, 3 H, H-14).

^{13}C δ = 172.9 (C-19), 172.4 (C-12), 171.1(C-16), 170.2 (C-23), 136.7 (C-38), 127.7 (C-43), 123.8(C-36), 121.0 (C-40), 118.6 (C-41), 118.4 (C-42), 111.3 (C-39), 110.3 (C-35), 54.4 (C-17), 50.0 (C-20), 49.0 (C-13), 31.4 (C-10), 30.3 (C-9), 27.0 (C-34), 26.1 (25 and 24), 17.1 (C-14), 16.8 (C-21).

(2*H*,2*H*,3*H*,3*H*-perfluoroundecanoyl)-Trp-Ala-Ala-(2*H*,2*H*,3*H*,3*H*-perfluorodecylamid) **7**

Identical procedure as for (2H,2H,3H,3H-perfluorooctanoyl)-Trp-Trp-Ala-(2H,2H,3H,3H-perfluorodecylamid) but employing (2*H*,2*H*,3*H*,3*H*-perfluoroundecanoyl)-Trp-Ala-Ala instead of (2*H*,2*H*,3*H*,3*H*-perfluorooctanoyl)-Ala-Trp-Ala. White powder (615 mg, 0.486 mmol, 40 %).

UPLC-MS: Method 2, T_R = 9.51 min; m/z MS (ES+): 803.0 [30%], 1266.0 [20%, M + H^+], 1288.0 [100%, M + Na^+], 1303.9 [10%, M + K^+].

ESI-HRMS: calculated for $C_{38}H_{30}F_{34}N_5O_4^+$: 1266.1749; found: 1266.1741.

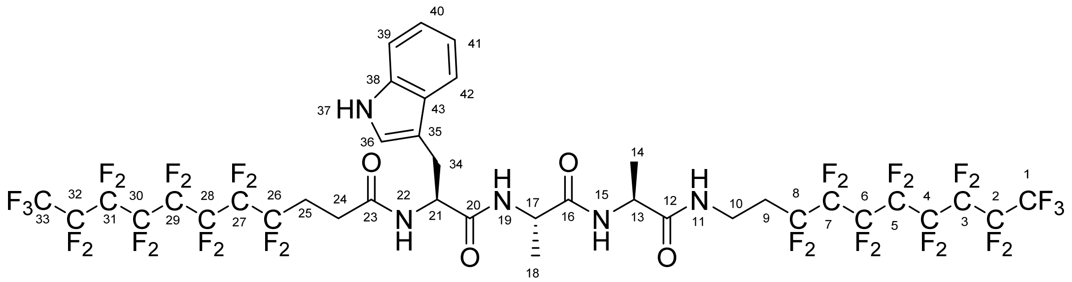

^{1}H-NMR (600 MHz, DMF-d$_7$, 333 K): δ = 10.70 (s, 1 H, H-37), 8.13 (d, $^3J_{H-H}$ = 6.9 Hz, 1 H, H-22), 7.96 (d, $^3J_{H-H}$ = 6.7 Hz, 1 H, H-19), 7.77 (t, $^3J_{H-H}$ = 5.7 Hz, 1 H, H-11), 7.63 (d, $^3J_{H-H}$ = 7.7 Hz, 1 H, H-15), 7.62 (d, $^3J_{H-H}$ = 7.8 Hz, 1 H, H-42), 7.40 (d, $^3J_{H-H}$ = 8.1 Hz, 1 H, H-39), 7.28 (d, $^3J_{H-H}$ = 2.3 Hz, 1 H, H-36), 7.09 (ddd, $^3J_{H-H}$ = 8.1 Hz, $^3J_{H-H}$ = 6.9 Hz, $^4J_{H-H}$ = 1.1 Hz, 1 H, H-40), 7.01 (ddd, $^3J_{H-H}$ = 8.0 Hz, $^3J_{H-H}$ = 6.9 Hz, $^4J_{H-H}$ = 1.0 Hz, 1 H, H-41), 4.67 (td, $^3J_{H-H}$ = 7.6 Hz, $^3J_{H-H}$ = 5.3 Hz, 1 H, H-21), 4.33 (p, $^3J_{H-H}$ = 7.1 Hz, 1 H, H-13), 4.32 (p, $^3J_{H-H}$ = 7.1 Hz, 1 H, H-17), 3.56-3.51 (m, 2 H, H-10), 3.32 (dd, $^2J_{H-H}$ = 14.8 Hz, $^3J_{H-H}$ = 5.2 Hz, 1 H, H-34), 3.18 (dd, $^2J_{H-H}$ = 14.8 Hz, $^3J_{H-H}$ = 8.0 Hz, 1 H, H-34), 2.67-2.48 (m, 6 H, H-24, H-25 and H-9), 1.31 (d, $^3J_{H-H}$ = 7.1 Hz, 3 H, H-21), 1.25 (d, $^3J_{H-H}$ = 7.1 Hz, 3 H, H-14).

^{13}C δ = 172.3 (C-38), 171.9 (C-16 and C-20), 170.3 (C-23), 136.7 (C-38), 127.8 (C-43), 123.8 (C-36), 120.9 (C-40), 118.3 (C-41 and C-42), 111.3 (C-39), 110.2 (C-35), 55.1 (C-21), 49.4 (C-17), 48.8 (C-13), 31.5 (C-10), 30.5 (C-9), 27.4 (C-37), 26.3 (C-24 and C-25), 17.2 (C-14), 16.8 (C-18).

(2*H*,2*H*,3*H*,3*H*-perfluoroundecanoyl)-Ala-Ala-Trp-(2*H*,2*H*,3*H*,3*H*-perfluorodecylamid) **8**

Identical procedure as for (2H,2H,3H,3H-perfluorooctanoyl)-Ala-Trp-Ala-(2H,2H,3H,3H-perfluorodecylamid) but employing (2*H*,2*H*,3*H*,3*H*-perfluoroundecanoyl)-Ala-Ala-Trp instead of (2*H*,2*H*,3*H*,3*H*-perfluorooctanoyl)-Ala-Trp-Ala. White powder (1.12 g, 0.885 mmol, 73 %).

UPLC-MS: Method 2, T_R = 9.60 min; m/z MS (ES+): 650.0 [20%], 1265.9 [30%, M + H$^+$], 1288.0 [100%, M + Na$^+$].

ESI-HRMS: calculated for $C_{38}H_{30}F_{34}N_5O_4^+$: 1266.1749; found: 1266.1745.

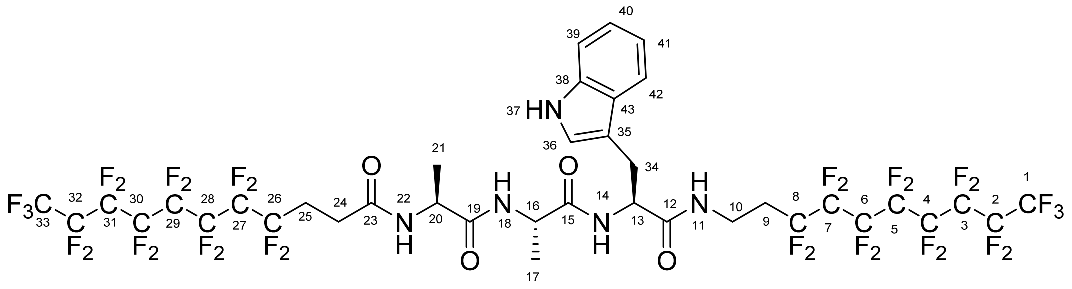

^{1}H-NMR (600 MHz, DMF-d$_7$, 333 K): δ = 10.67 (s, 1 H, H-37), 8.17 (d, $^3J_{H-H}$ = 6.5 Hz, 1 H, H-22), 8.00 (d, $^3J_{H-H}$ = 7.6 Hz, 1 H, H-18), 7.73 (t, $^3J_{H-H}$ = 5.7 Hz, 1 H, H-11), 7.61 (d, $^3J_{H-H}$ = 7.9 Hz, 1 H, H-42), 7.60 (d, $^3J_{H-H}$ = 7.9 Hz, 1 H, H-14), 7.38 (d, $^3J_{H-H}$ = 8.1 Hz, 1 H, H-39), 7.20 (d, $^3J_{H-H}$ = 2.3 Hz, 1 H, H-36), 7.08 (t, $^3J_{H-H}$ = 7.5 Hz, 1 H, H-40), 7.00 (t, $^3J_{H-H}$ = 7.4 Hz, 1 H, H-41), 4.60 (td, $^3J_{H-H}$ = 7.6 Hz, $^3J_{H-H}$ = 5.7 Hz, 1 H, H-13), 4.36 (dq, $^3J_{H-H}$ = 7.0 Hz, 1 H, H-20), 4.30 (dq, $^3J_{H-H}$ = 7.0 Hz, 1 H, H-16), 3.53-3.41 (m, 2 H, H-10), 3.28 (dd, $^2J_{H-H}$ = 14.6 Hz, $^3J_{H-H}$ = 5.8 Hz, 1 H, H-34), 3.18 (dd, $^2J_{H-H}$ = 14.6 Hz, $^3J_{H-H}$ = 7.6 Hz, 1 H, H-34), 2.66-2.55 (m, 4 H, H-24 and H-25), 2.42-2.34 (m, 2 H, H-9), 1.30 (d, $^3J_{H-H}$ = 7.1 Hz, 3 H, H-21), 1.27(d, $^3J_{H-H}$ = 7.1 Hz, 3 H, H-17).

^{13}C δ = 172.7 (C-19), 172.0 (C-15), 171.6 (C-12), 170.0 (C-23), 136.8 (C-38), 127.8(C-43), 123.6 (C-36), 121.0 (C-40), 118.5 (C-42), 118.3 (C-41), 111.2 (C-39), 110.4 (C-35), 54.2 (C-13), 49.6 (C-20), 49.5 (C-16), 31.5 (C-10), 30.5 (C-9), 28.5 (C-34), 26.3 (C-24 and C-25), 17.0 (C-21), 16.9 (C-17).

NHS-ester of *2H,2H,3H,3H*-perfluorodecanoic acid[3]

2H,2H,3H,3H-perfluorodecanoic acid (1.00 eq., 15.0 g, 30.5 mmol) was dissolved in THF (500 ml), then HSTU (1.50 eq., 13.8 g, 45.8 mmol) and DIPEA (1.49 eq., 7.50 ml, 45.4 mmol) were added and the solution was stirred overnight. The solvent was removed under reduced pressure and the crude product dissolved in EtOAc (1 l) and washed with 0.1 M HCl (2 $\times$ 200 ml), brine (200 ml) and H_2O (200 ml). After removal of volatiles under reduced pressure the crude product was purified by column chromatography (EtOAc/cyclohexane (1:1)). The product was obtained as a white solid (16.1 g, 27.4 mmol, 90 %).

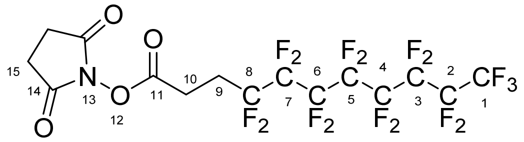

^{1}H-NMR (CDCl$_3$, 500 MHz, 293 K): δ = 3.03 – 2.92 (m, 2 H, H-10), 2.86 (s, 4 H, H-15), 2.68-2.45 (m, 2 H, H-9).

^{13}C δ = 168.7 (C-14), 166.7 (C-11), 117.3(C-8), 26.2(C-9), 25.5 (C-15), 22.8 (C-10).

Trp-Lys-Trp-Lys-Trp-Lys-Trp-Lys-Trp

2-chlorotrityl chloride resin (200 mg) was placed in a plastic syringe equipped with a filter frit. The CH_2Cl_2 was exchanged and DIPEA (2.4 eq, 20 µl, 0.12 mmol), and Fmoc-Trp(boc)-OH (1.00 eq., 26.3 mg, 49.9 µmol) were added. After the sample was agitated on a shaker for 2.5 h liquids were removed by filtration and the resin washed with CH_2Cl_2 (~4 ml) and DMF (~4 ml). The unreacted binding sites on the resin were capped by adding MeOH-solution (~5 ml, 15% MeOH, 5% DIPEA, 80% CH_2Cl_2) followed by agitation for 30 minutes. The peptide coupling steps were performed using a (intavis multi pep RSI) peptide synthesizer and employing 5.00 eq. of Fmoc-amino acids, PyBOP and DIPEA with a reaction time of first

45 min followed be a 2nd coupling step for 90 min. Fmoc-deprotection was carried out with piperidine (20 %, 4 × 5 min). In between coupling and Fmoc-deprotection the syringes were washed 5 times with DMF. In the end the syringe was washed with CH_2Cl_2 (5 × ~2 ml) before the cleavage solution (5ml, 96 % TFA, 3 % triisopropylsilane, 3 % H_2O) was added. Cleavage, precipitation and washing (Et_2O) were performed as described above. The product was isolated as a white solid (64 mg, 44 μmol, 88 %).

UPLC-MS: Method 1, T_R = 4.82 min; m/z MS (ES+): 488.4 [100 %, M + $3H^+$],731.4 [60 %, M + $2H^+$],1462.3 [25%, M + H^+].

ESI-HRMS: calculated for : $C_{79}H_{103}N_{18}O_{10}^{3+}$: 487.9363; found: 487.9367.

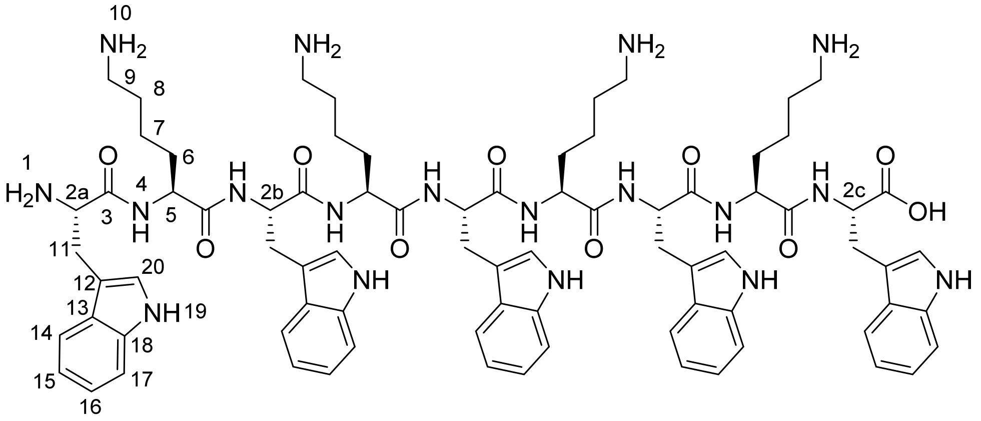

^{1}HNMR (DMSO, 500 MHz, 293 K): δ = 10.94 (s, 1 H, H-19), 10.82 (s, 1 H, H-19),10.78-10.71 (m, 3 H, H-19), 8.20-7.90 (m, 8 H, H-4), 7.90-7.70 (b, 10 H, H-1 and H-10), 7.64 (d, $^3J_{H-H}$ = 7.9 Hz, 1 H, H-14), 7.60 (d, $^3J_{H-H}$ = 7.9 Hz, 1 H, H-14) 7.57 (d, $^3J_{H-H}$ = 8.0 Hz, 1 H, H-14), 7.56 (d, $^3J_{H-H}$ = 7.9 Hz, 1 H, H-14), 7.52 (d, $^3J_{H-H}$ = 8.0 Hz, 1 H, H-14), 7.35 (d, $^3J_{H-H}$ = 8.2 Hz, 1 H, H-17), 7.32-7.27 (m, 4 H, H-17), 7.17 (d, $^3J_{H-H}$ = 2.4, 1 H, H-20), 7.15- 7.09 (m, 4 H, H-20), 7.07-7.00 (m, 5 H, H-16), 6.99-6.86 (m, 5 H, H-15), 4.65-4.55 (m, 3 H, H-2b), 4.50-4.40 (m, 1 H, H-2c), 4.35-4.20 (m, 4 H, H-5), 4.02-3.93 (1 H, H-2a), 3.20-2.90 (m, 10 H, H-11), 2.75-2.65 (m, 8 H, H-9), 1.68-1.41 (m, 16 H, H-6 and H-8), 1.32-1.15 (m, 8 H, H-7).

^{13}C ((RR'*C*=O) not detected) δ = 136.5 (5 C, C-18), 127.8 (5 C, C-13), 125.5 (C-20), 124.1 (4 C, C-20), 121.4 (5 C, C-16), 119.0 (5 C, C-14), 118.7 (5 C, C-15)111.8 (5 C, C-17), 110.3 (C-12), 53.7 (4 C, C-2b and C-2c), 52.9 (5 C, C-5 and C-2a), 39.1 (4 C, C-9), 32.0 (4 C, C-6), 28.2 (5 C, C-11), 27.1 (4 C, C-8), 22.5 (4 C, C-8).

Compound **9**

Trp-Lys-Trp-Lys-Trp-Lys-Trp-Lys-Trp (1.00 eq., 120 mg, 82.1 μmol) and *1H,1H,2H,2H*-perfluorodecanoic acid NHS ester (10.0 eq., 482 mg, 0.821 mmol) were dissolved in DMF (200ml), then DIPEA (20 eq., 0.30 ml, 1.6 mmol) was added and the solution was stirred for 56 h at 50°C. Subsequently the mixture was concentrated to 25 mL under reduced pressure and water (175 ml) was added. The precipitate was collected and washed with acetone (3 × 50 ml). The product was obtained as a pale brown solid (245 mg, 64.0 μmol, 80 %).

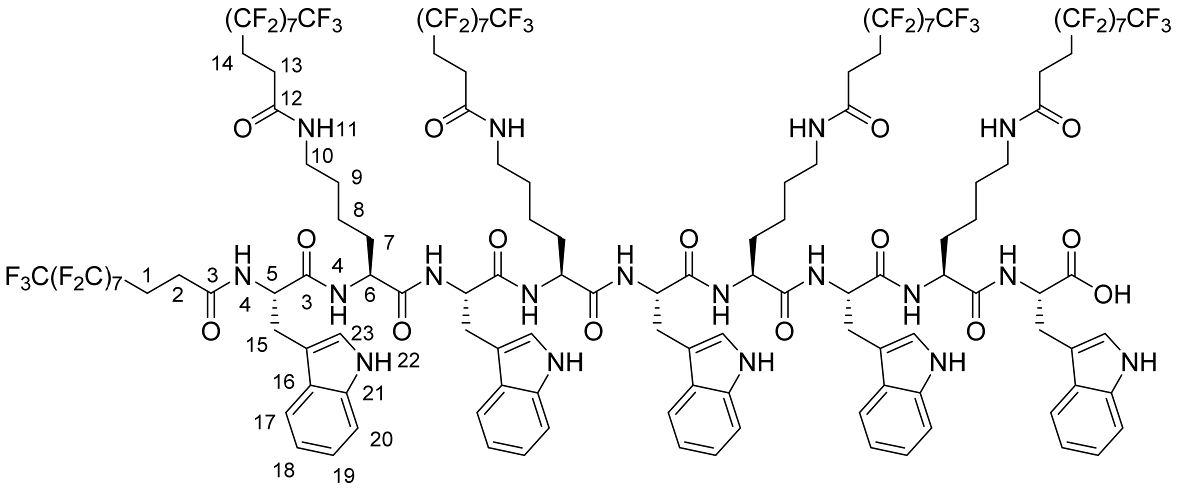

^{1}H NMR (HFIP-d_2 + 20 mol% H_2O, presat (water and OH-HFIP signal suppression, 500 MHz, 293 K): δ = 8.75 (s, 1 H, H-22), 8.74 (s, 1 H, H-22), 8.68 (s, 1 H, H-22), 8.65 (s, 1 H, H-22), 8.45 (s, 1 H, H-22), 7.66 (d, $^{3}J_{H-H}$ = 7.9 Hz, 1 H, H-17), 7.60 (d, $^{3}J_{H-H}$ = 8.2 Hz, 1H, H-17), 7.58 (d, $^{3}J_{H-H}$ = 8.3 Hz, 1 H, H-17), 7.54 (d, $^{3}J_{H-H}$ = 8.0 Hz, 1 H, H-17), 7.49 (d, $^{3}J_{H-H}$ = 7.9 Hz, 1 H, H-17), 7.46-7.37 (m, 4 H, H-17), 7.36 (s, 1 H, H-4), 7.33-7.12 (m, 13 H, 1 H, H-17, 5 H, H-18, 5 H, H-19, 2 H, H-4 (7.27 and 7.18 according to COSY), 7.10 (s, 1 H, H-23), 7.09 (s, 1 H, H-4), 7.08 (s, 1 H, H-23), 7.02 (s, 1 H, H-23), 7.01 (s, 1 H, H-23), 6.96 (s, 2 H, H-4), 6.90 (s, 1 H, H-23), 6.84 (s, 1 H, H-4), 6.56 (s, 1 H, H-4), 6.30-6.15 (m, 4 H, H-11), 4.82-4.76 (m, 1 H, H-5), 4.65-4.58 (m, 2 H,H-5), 4.58-4.50 (m, 2 H, H-5), 4.10 (t, $^{3}J_{H-H}$ = 6.9 Hz, 1 H, H-6), 4.05-3.98 (m, 2 H, H-6), 3.94-3.89 (m, 1 H, H-6), 3.50-2.95 (m,18 H, H-15 and H-10), 2.60-2.25 (m, 20 H, H-1 and H-2), 1.77-1.41 (m, 8 H, H-7), 1.41-1.25 (m, 8 H, H-8), 1.16-0.86 (m, 8 H, H-9).

^{13}C (no HMBC experiment was recorded - carbon atoms without hydrogen substituents were not detected) δ = 123.0 (C-23), 122.7 (C-23), 122.4 (3 C, C-23), 121.4 (5 C, C-19), 118.7 (5 C, C-18), 117.2 (C-17), 116.9 (2 C, C-17), 116.7 (2 C, C-17), 110.6 (5 C, C-20), 54.7 (C-6), 54.6 (C-6), 54.3 (C-6), 53.9 (2 C, C-5), 53.6 (2 C, C-5), 53.4 (C-6), 52.5 (C-5), 38.4 (4 C, C-10), 29.2 (4 C, C-7), 26.8 (C-8), 25.6 (10 C, C-1 and C-2), 25.2 (5 C, C-15), 21.2 (C-9).

MALDI: 3853.5 [M + Na^{+}].

^{1}H NMR spectra and LC chromatograms

Ala-Trp-Ala **1**

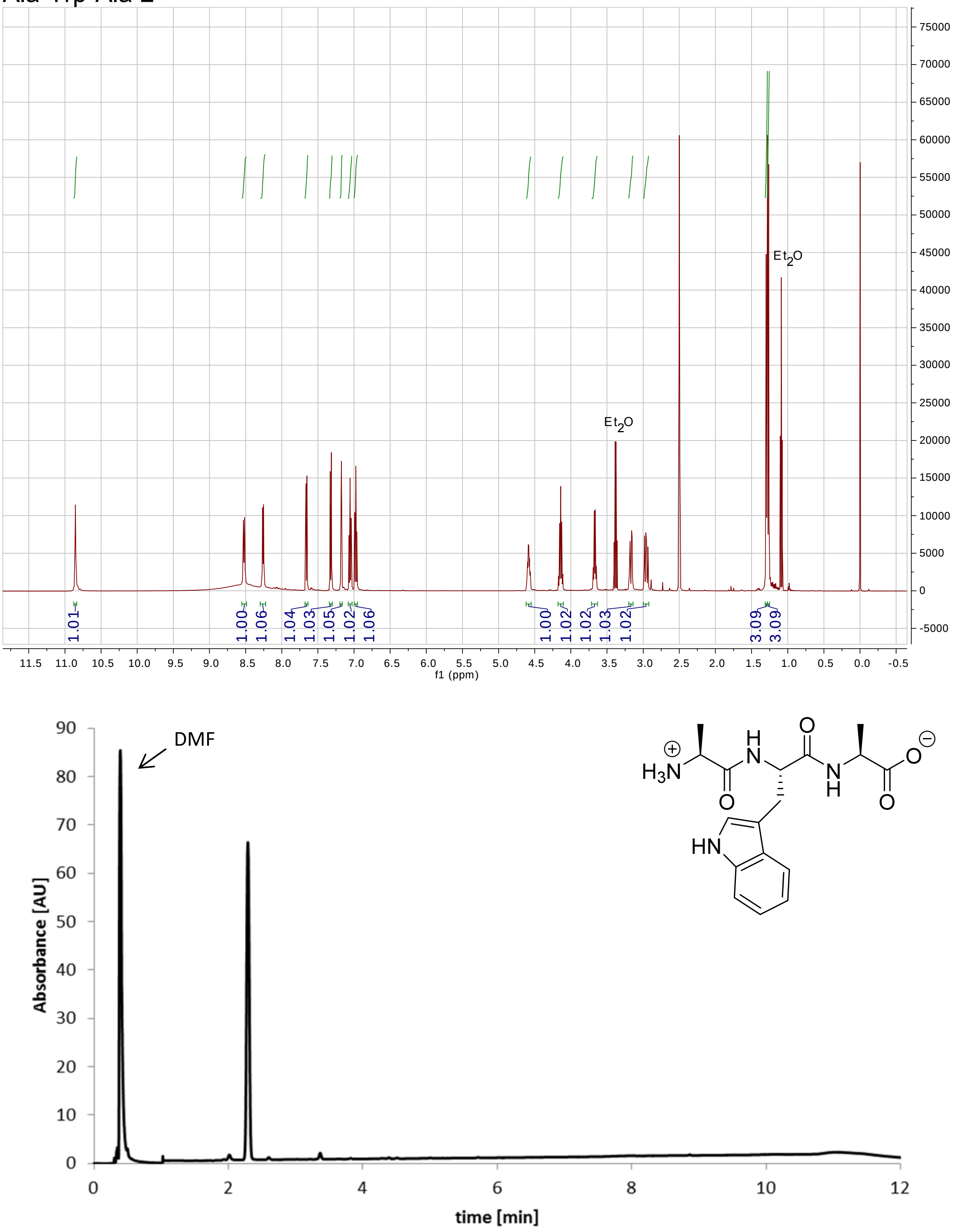

S1. Ala-Trp-Ala **1**. Top: ^{1}H-NMR (DMSO-d$_6$, 500 MHz, 293 K), Bottom: UV-Vis-trace 190-500 nm of UPLC chromatogram (Method 1).

Ac-Ala-Trp-Ala-NH2

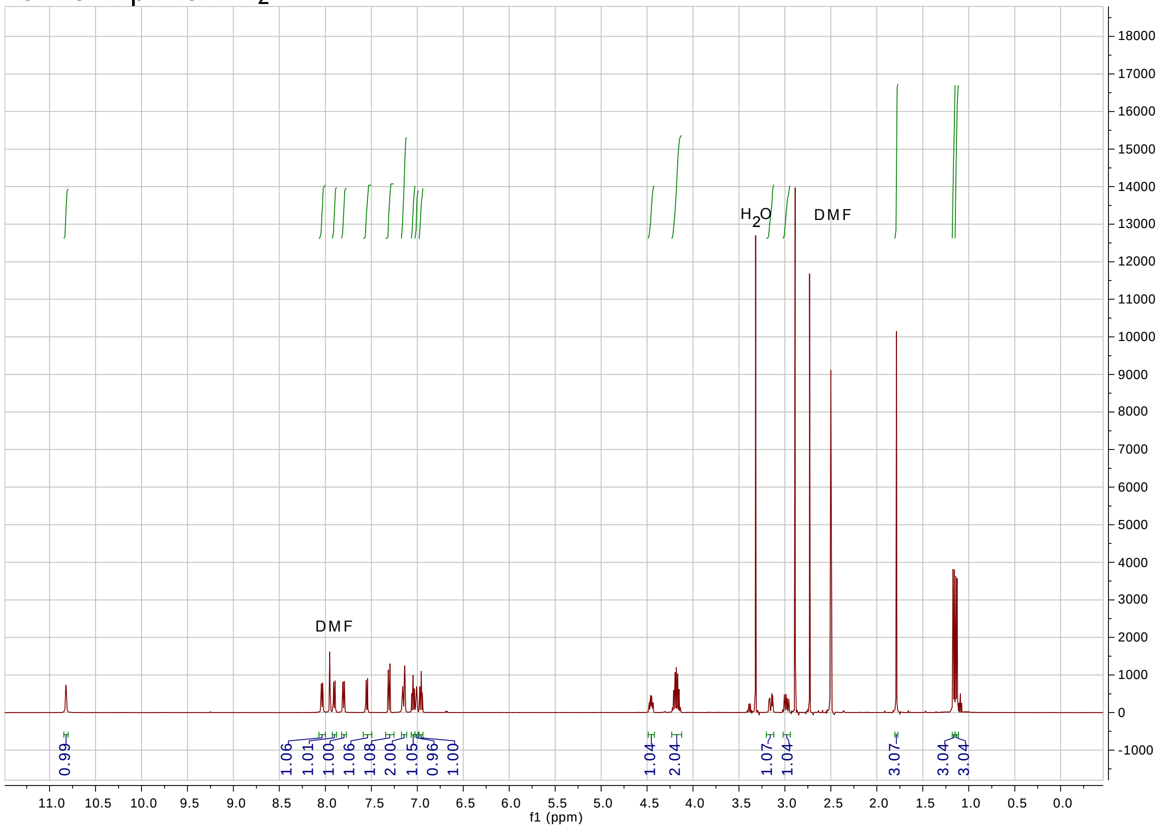

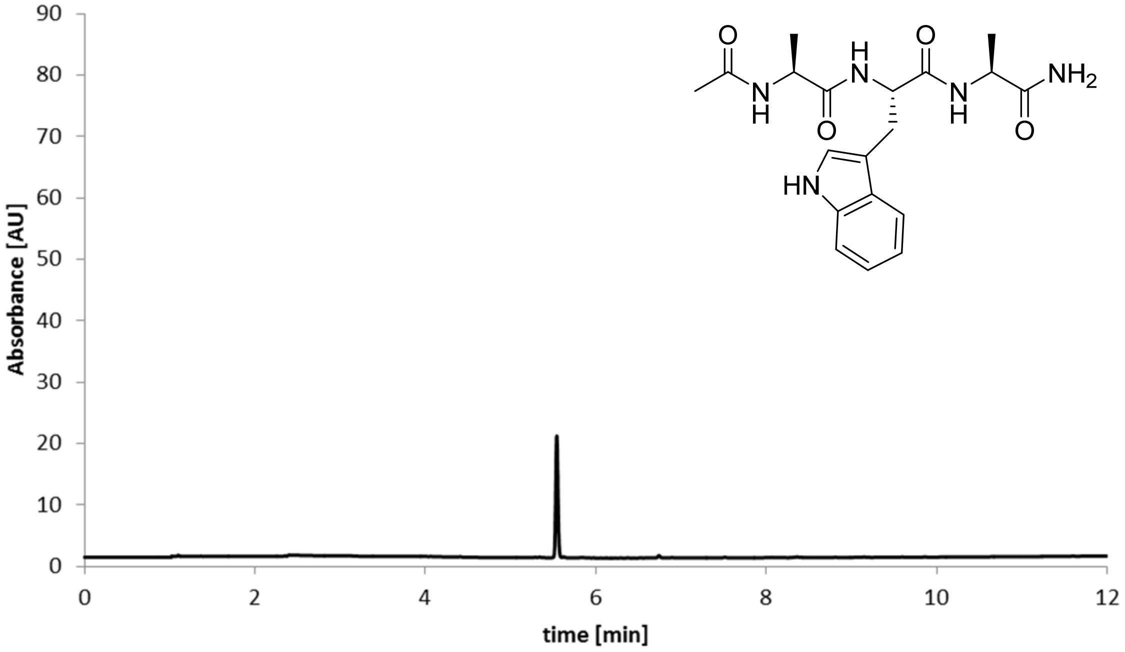

S2. Ac-Ala-Trp-Ala-NH_2. Top: ^{1}H-NMR (DMSO-d_6, 500 MHz, 293 K), Bottom: UV-Vis-trace 190-500 nm of UPLC chromatogram (Method 1).

(2*H*,2*H*,3*H*,3*H*-perfluorooctanoyl)-Ala-Trp-Ala

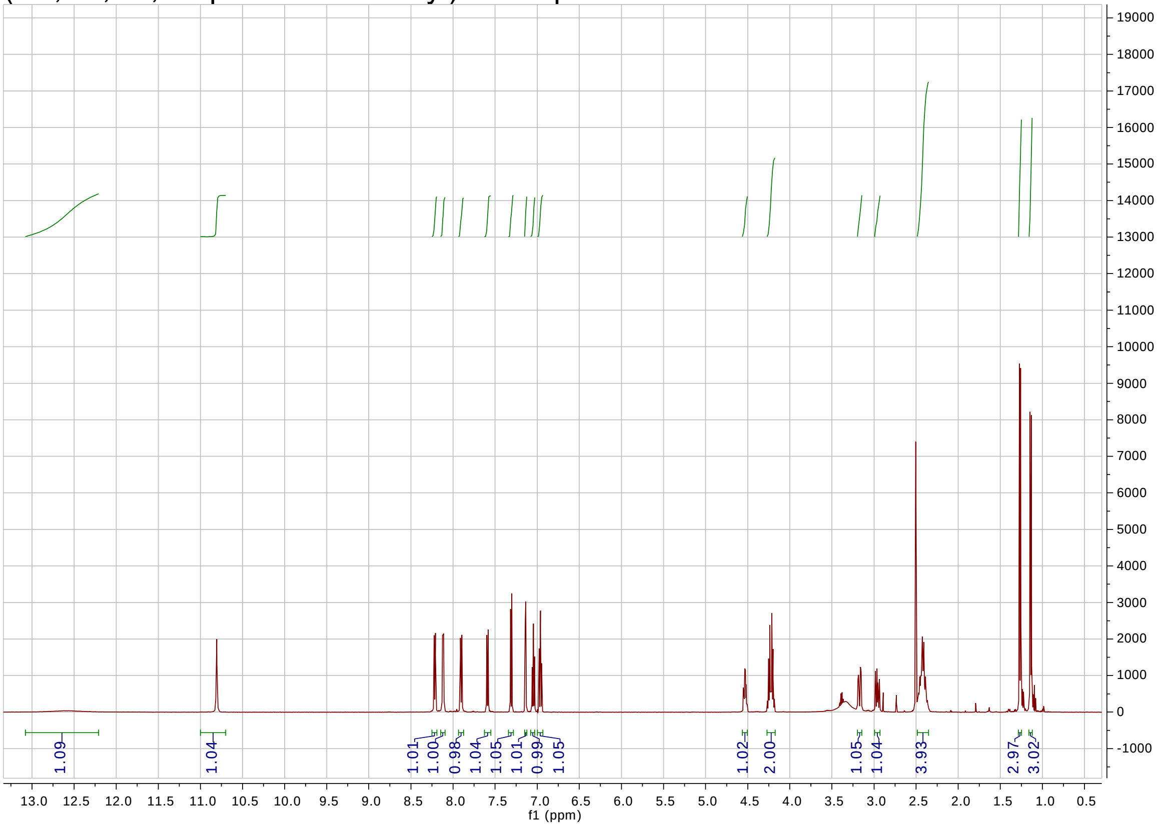

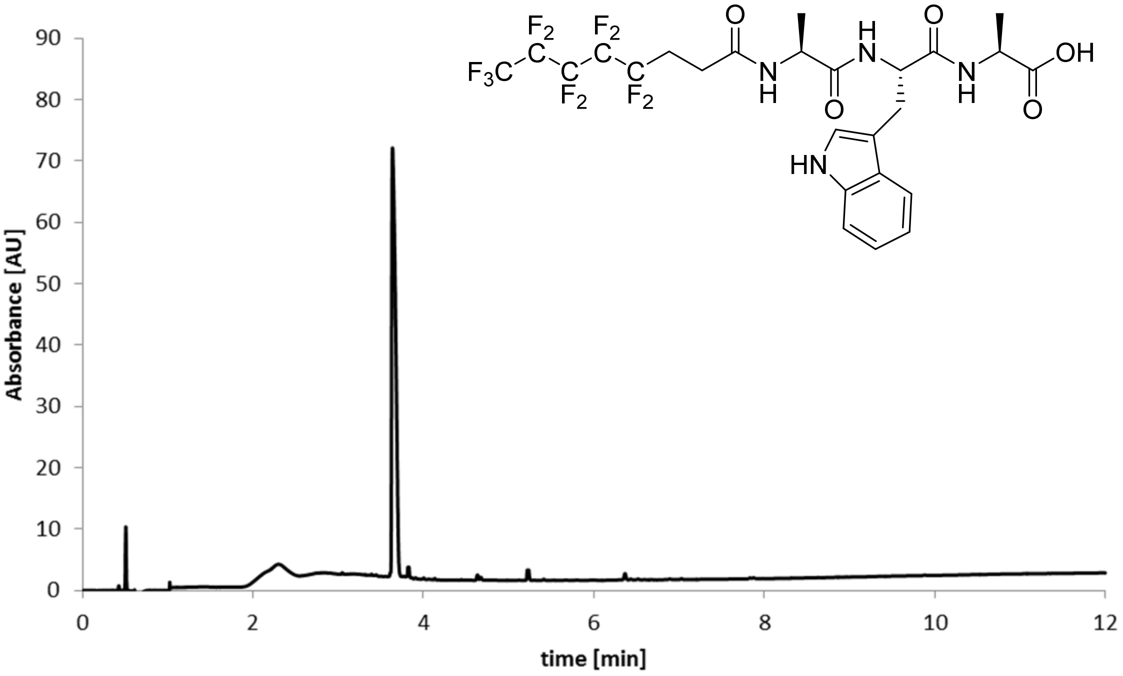

S3. (2*H*,2*H*,3*H*,3*H*-perfluorooctanoyl)-Ala-Trp-Ala. Top: ^{1}H-NMR (DMSO-d_6, 500 MHz, 293 K), Bottom: UV-Vis-trace 190-500 nm of UPLC chromatogram (Method 2).

(2*H*,2*H*,3*H*,3*H*-perfluoroundecanoyl)-Ala-Trp-Ala

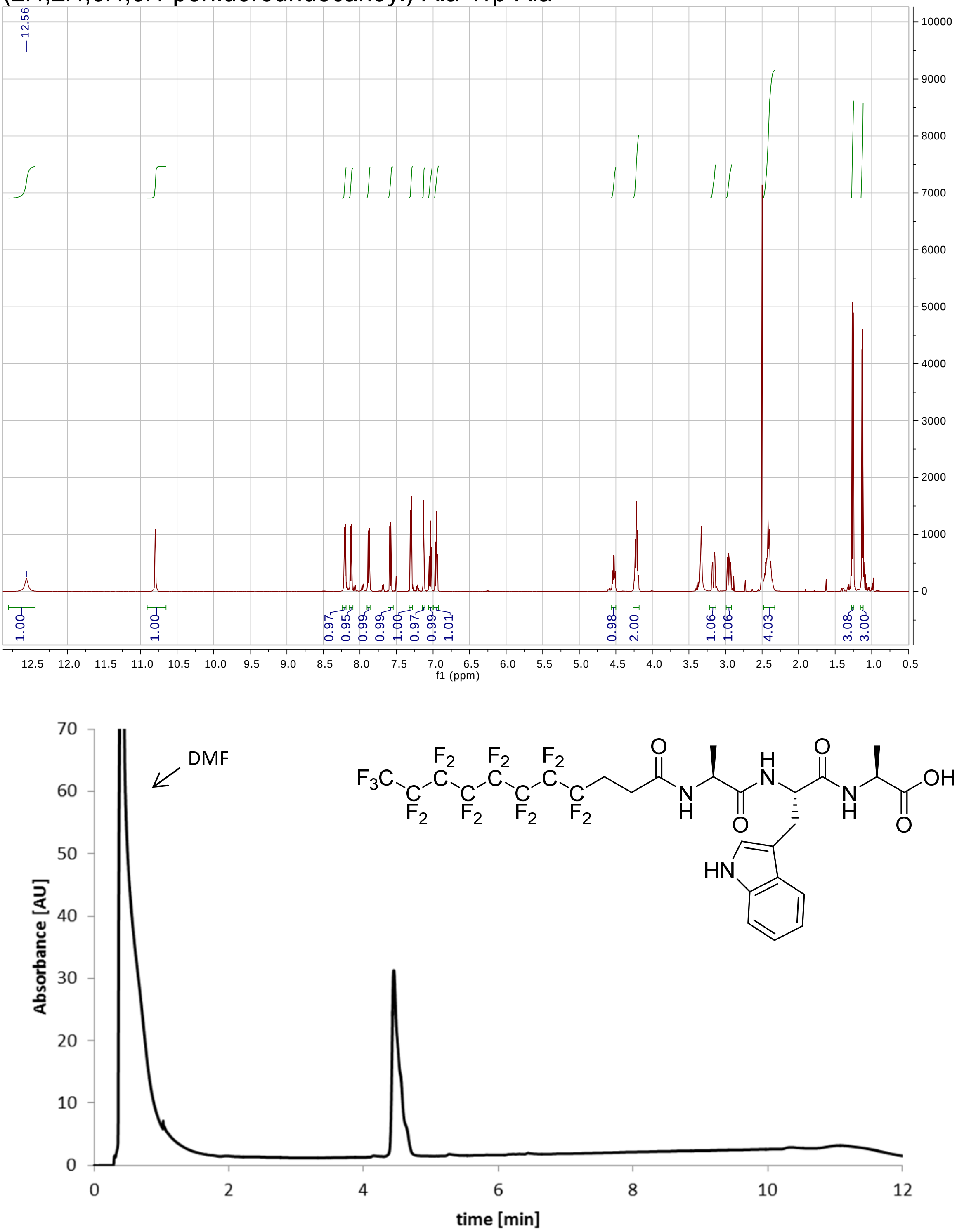

S4. (2*H*,2*H*,3*H*,3*H*-perfluoroundecanoyl)-Ala-Trp-Ala. Top: ^{1}H-NMR (DMSO-d_6, 500 MHz, 293 K), Bottom: UV-Vis-trace 190-500 nm of UPLC chromatogram (Method 2).

(2*H*,2*H*,3*H*,3*H*-perfluoroundecanoyl)-Trp-Ala-Ala

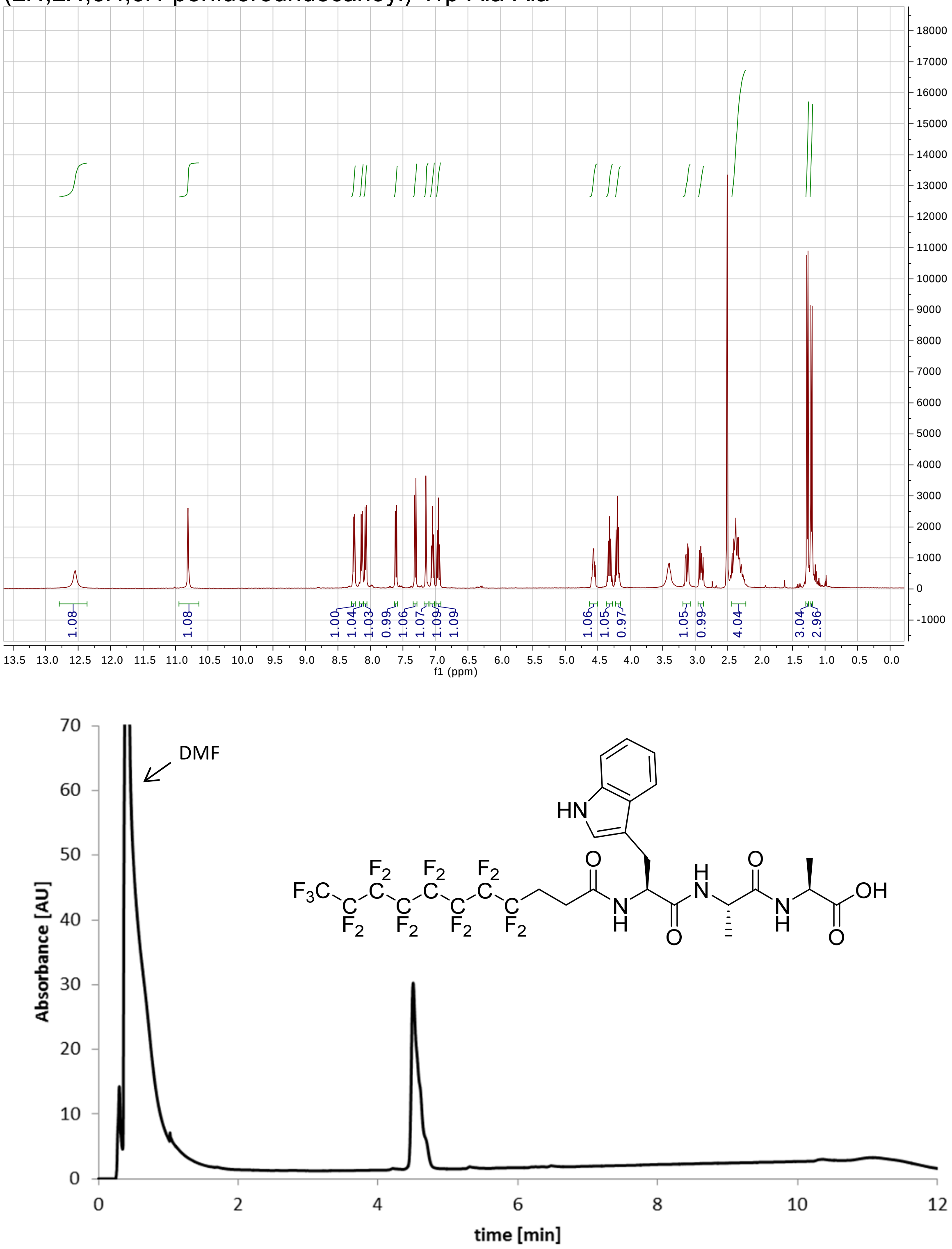

S5. (2*H*,2*H*,3*H*,3*H*-perfluoroundecanoyl)-Trp-Ala-Ala: Top: ^{1}H-NMR (DMSO-d_6, 500 MHz, 293 K), Bottom: UV-Vis-trace 190-500 nm of UPLC chromatogram (Method 2).

(2*H*,2*H*,3*H*,3*H*-perfluoroundecanoyl)-Ala-Ala-Trp

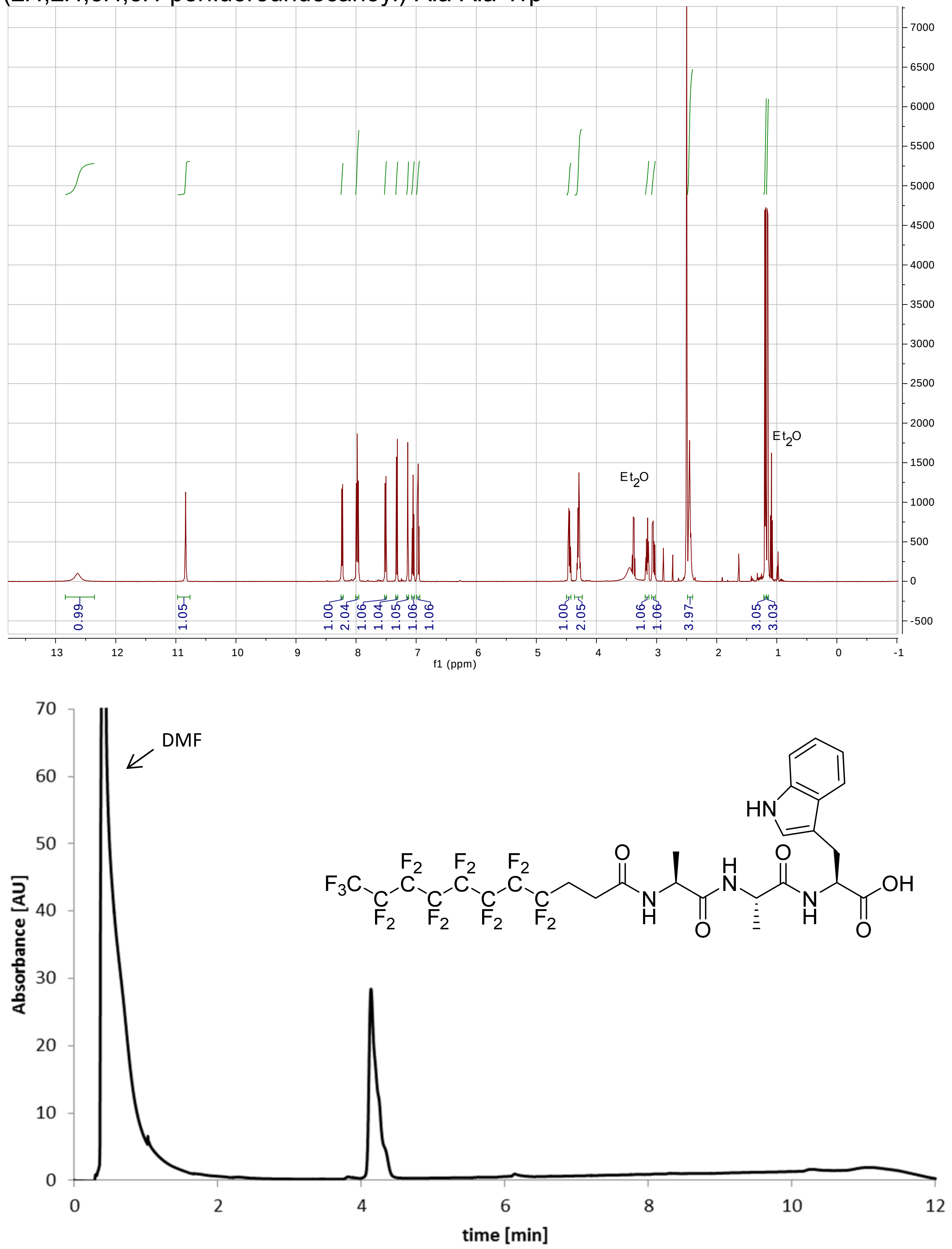

S6. (2*H*,2*H*,3*H*,3*H*-perfluoroundecanoyl)-Ala-Ala-Trp: Top: ^{1}H-NMR (DMSO-d_6, 500 MHz, 293 K), Bottom: UV-Vis-trace 190-500 nm of UPLC chromatogram (Method 2).

Ac-N^{α}-Me-Ala-N^{α}-Me-Trp(Me)-N^{α}-Me-Ala-N(Me)$_2$ **2**

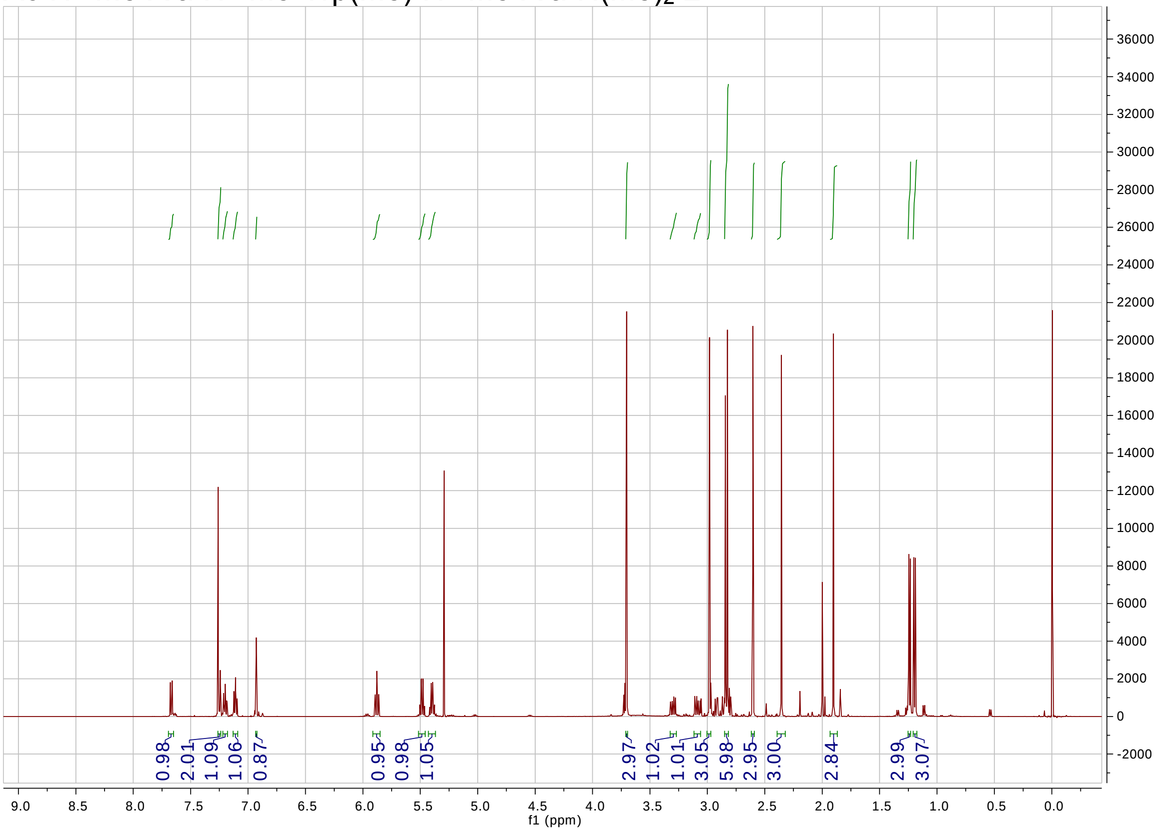

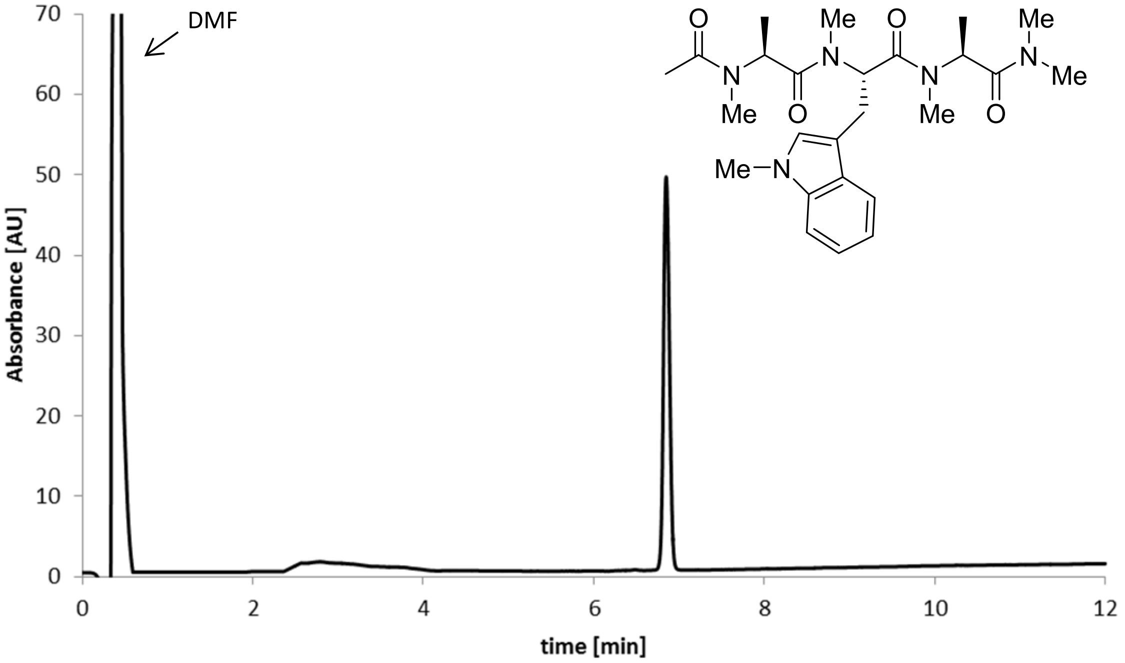

S7. Ac-N$^{\alpha}$-Me-Ala-N$^{\alpha}$-Me-Trp(Me)-N$^{\alpha}$-Me-Ala-N(Me)$_2$ **2**. Top: ^{1}H-NMR (CDCl$_3$, 500 MHz, 293 K), Bottom: UV-Vis-trace 190-500 nm of UPLC chromatogram (Method 2).

(2*H*,2*H*,3*H*,3*H*-perfluorooctanoyl)-Ala-Trp-Ala-NH_2 **3**

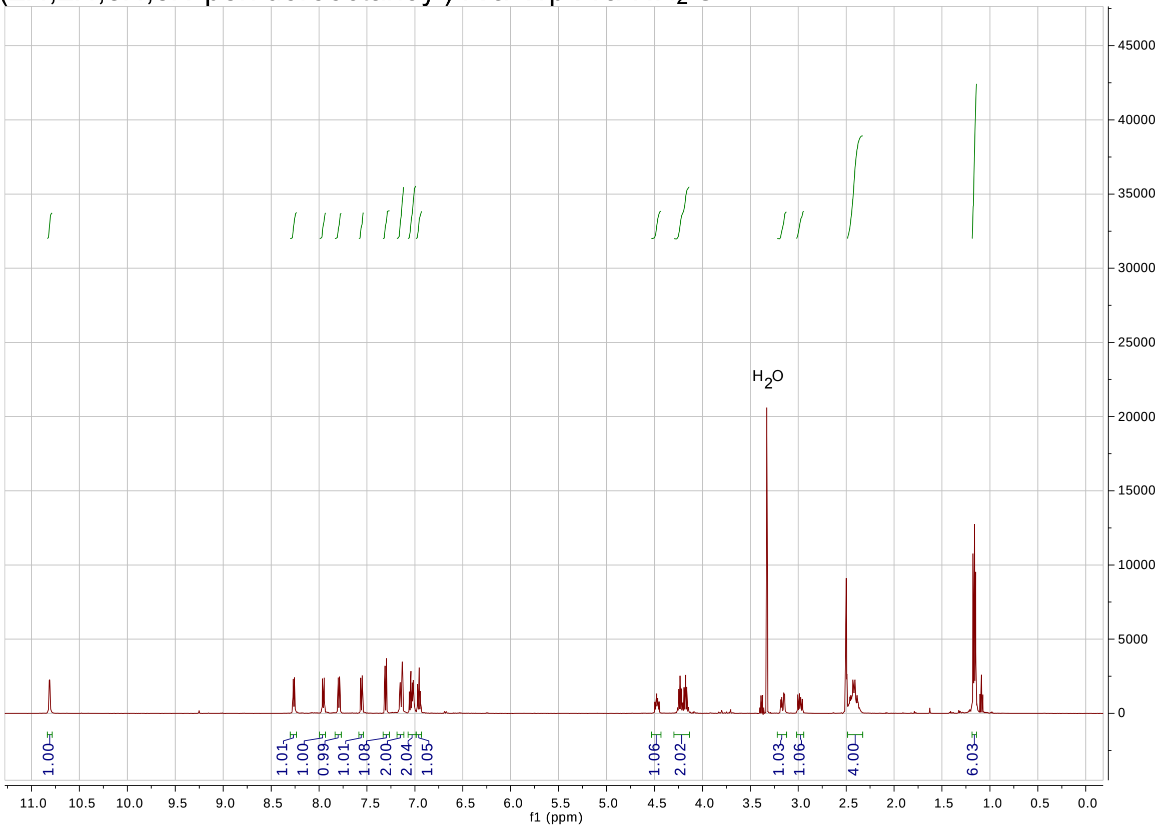

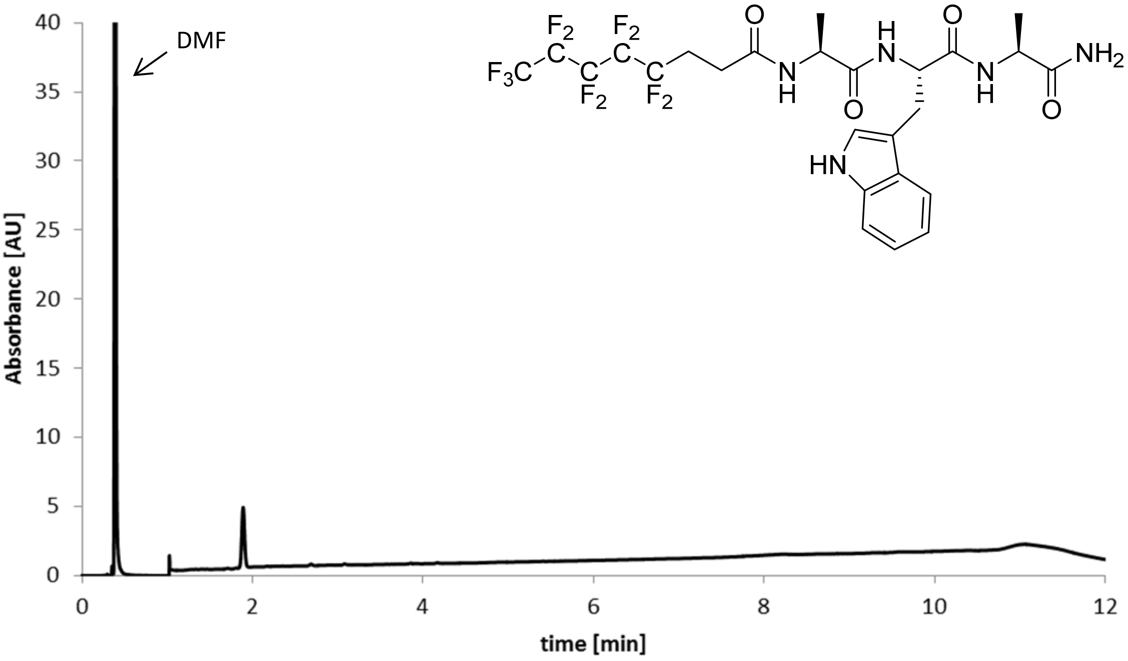

S8. (2*H*,2*H*,3*H*,3*H*-perfluorooctanoyl)-Ala-Trp-Ala-NH_2. Top: ^{1}H-NMR (DMSO-d_6, 500 MHz, 293 K), Bottom: UV-Vis-trace 190-500 nm of UPLC chromatogram (Method 2).

(2*H*,2*H*,3*H*,3*H*-perfluorooctanoyl)-N^{α}-Me-Ala-N^{α}-Me-Trp(Me)-N^{α}-Me-Ala-N(Me)$_2$ **4**

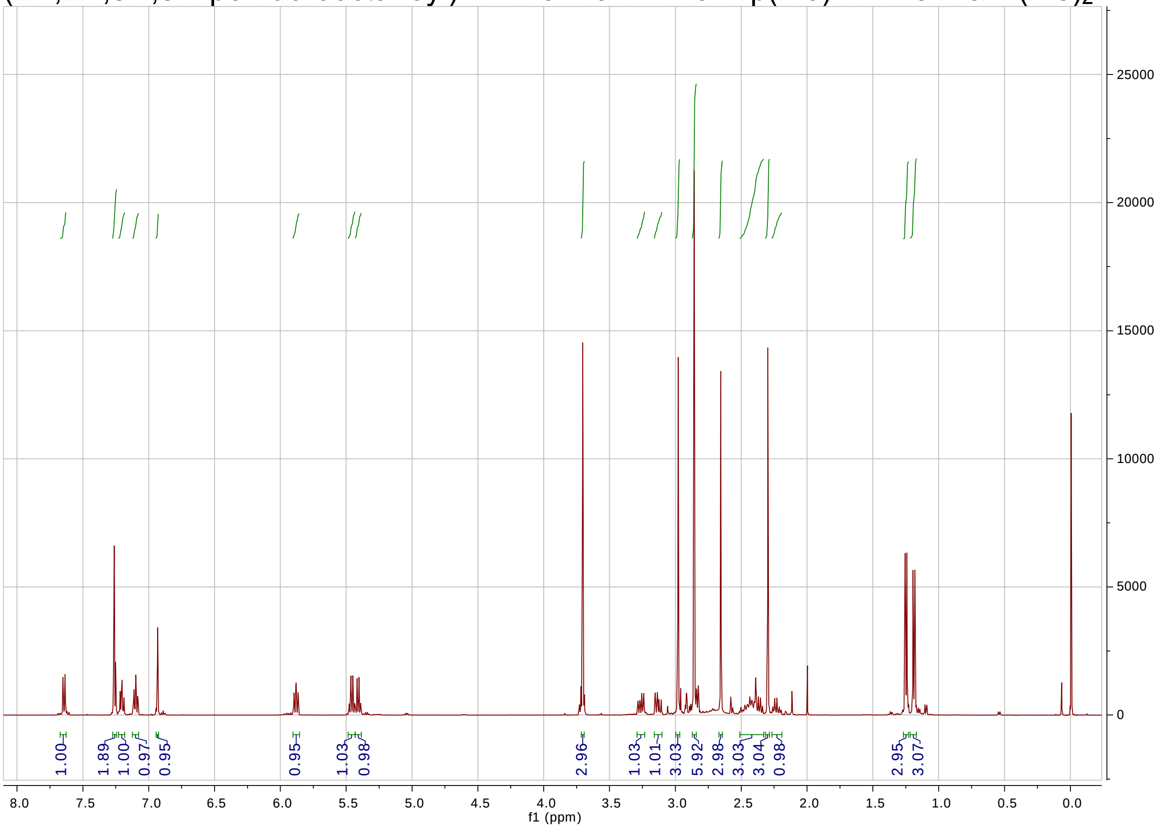

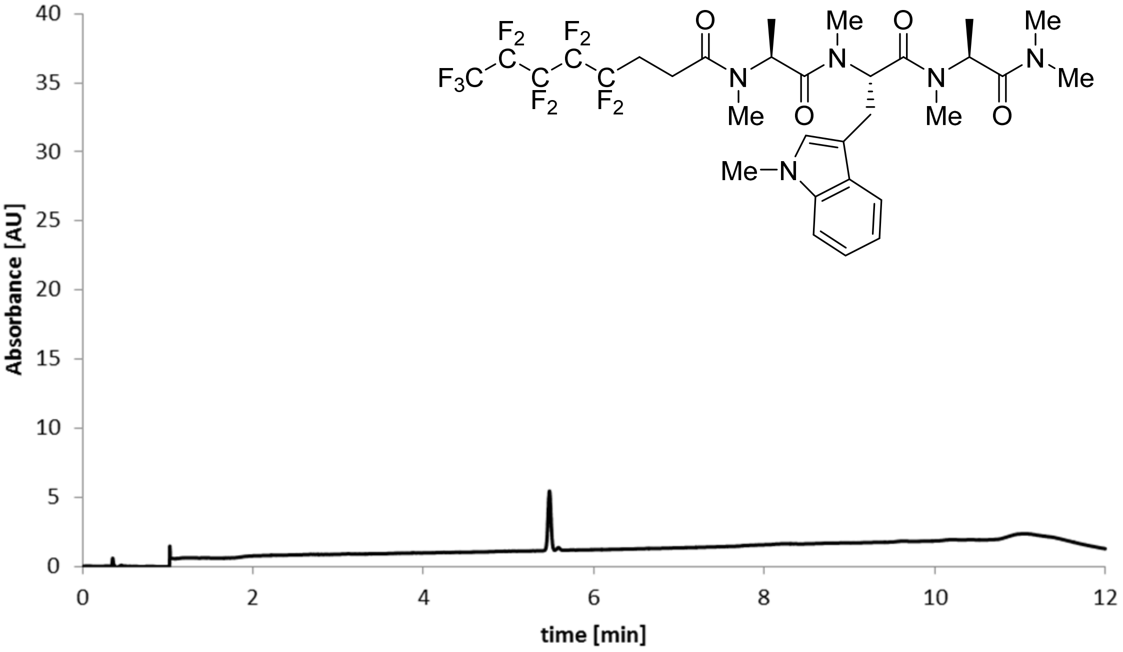

S9. (2*H*,2*H*,3*H*,3*H*-perfluorooctanoyl)-N^{α}-Me-Ala-N^{α}-Me-Trp(Me)-N^{α}-Me-Ala-N(Me)$_2$. Top: ^{1}H-NMR ($CDCl_3$, 500 MHz, 293 K), Bottom: UV-Vis-trace 190-500 nm of UPLC chromatogram (Method 2).

(2*H*,2*H*,3*H*,3*H*-perfluorooctanoyl)-Ala-Trp-Ala-(2*H*,2*H*,3*H*,3*H*-perfluorodecylamid) **5**

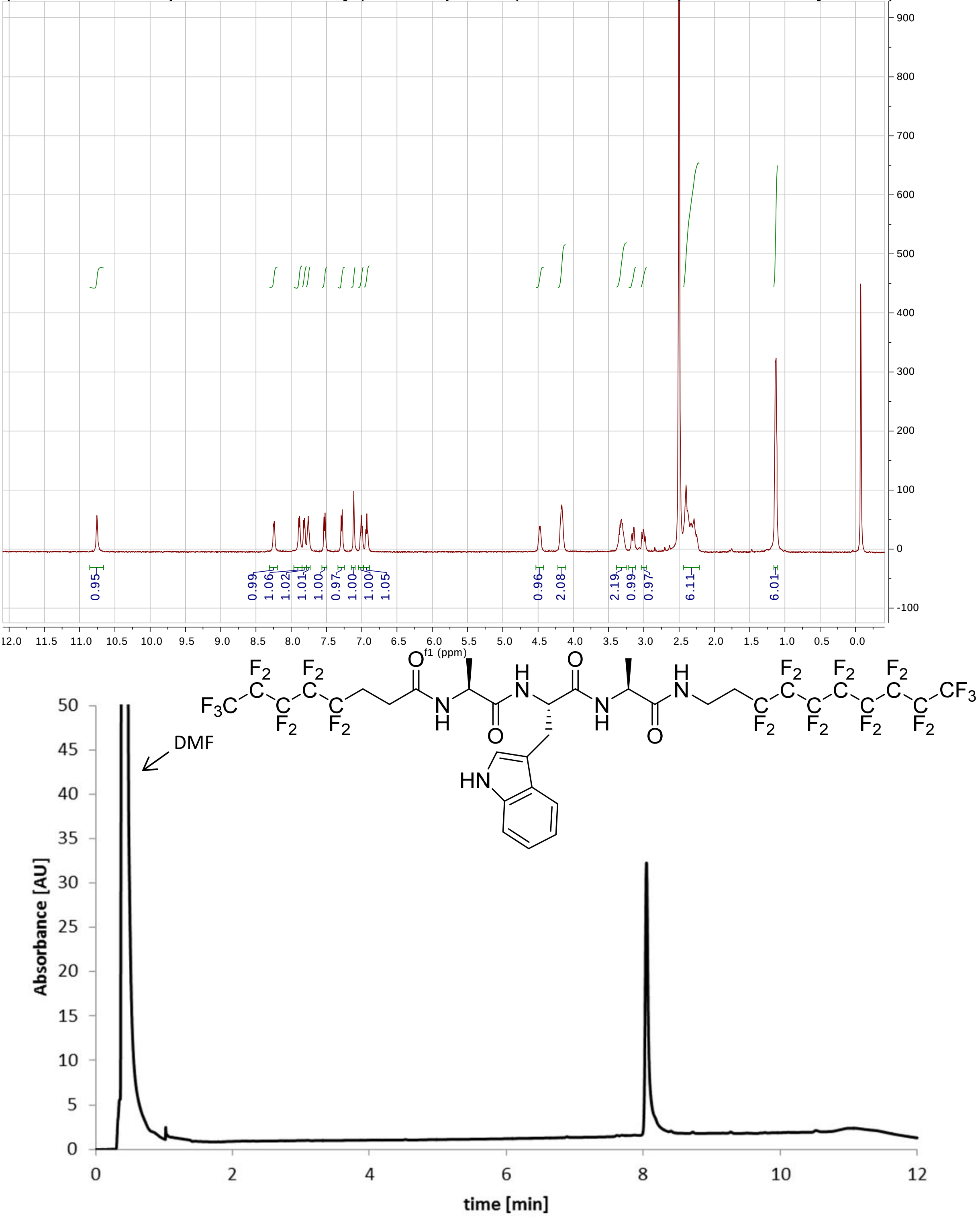

S9:

S10. (2*H*,2*H*,3*H*,3*H*-perfluorooctanoyl)-Ala-Trp-Ala-(2*H*,2*H*,3*H*,3*H*-perfluorodecylamid). Top: ^{1}H-NMR (DMSO-d_6, 500 MHz, 293 K), Bottom: UV-Vis-trace 190-500 nm of UPLC chromatogram (Method 2).

(2*H*,2*H*,3*H*,3*H*-perfluoroundecanoyl)-Ala-Trp-Ala-(2*H*,2*H*,3*H*,3*H*-perfluorodecylamid) **6**

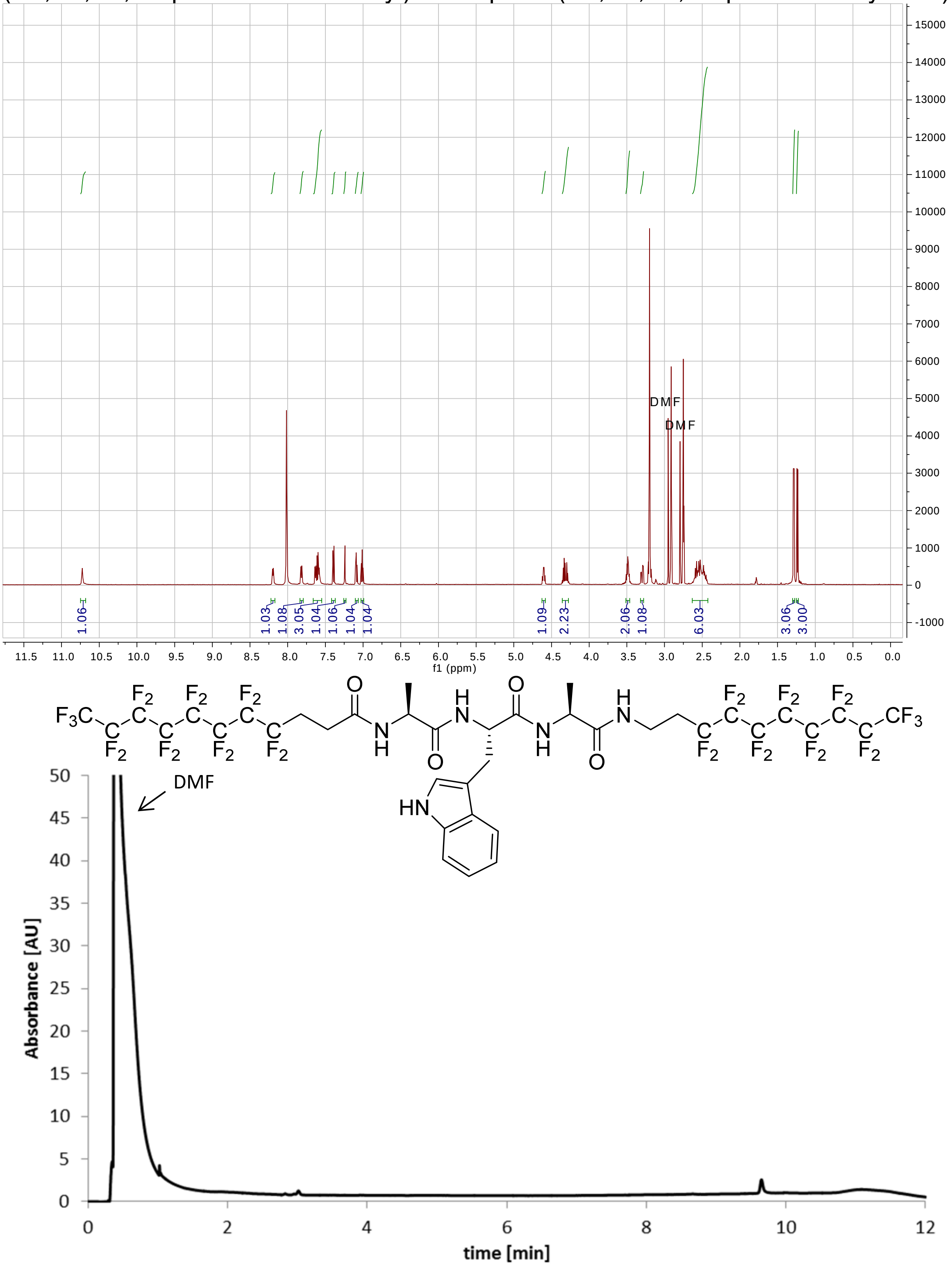

S11. (2*H*,2*H*,3*H*,3*H*-perfluoroundecanoyl)-Ala-Trp-Ala-(2*H*,2*H*,3*H*,3*H*-perfluorodecylamid) **6**. Top: ^{1}H-NMR (DMF-d_7, 600 MHz, 333 K), Bottom: UV-Vis-trace 190-500 nm of UPLC chromatogram (Method 2).

(2*H*,2*H*,3*H*,3*H*-perfluoroundecanoyl)-Trp-Ala-Ala-(2*H*,2*H*,3*H*,3*H*-perfluorodecylamid) **7**

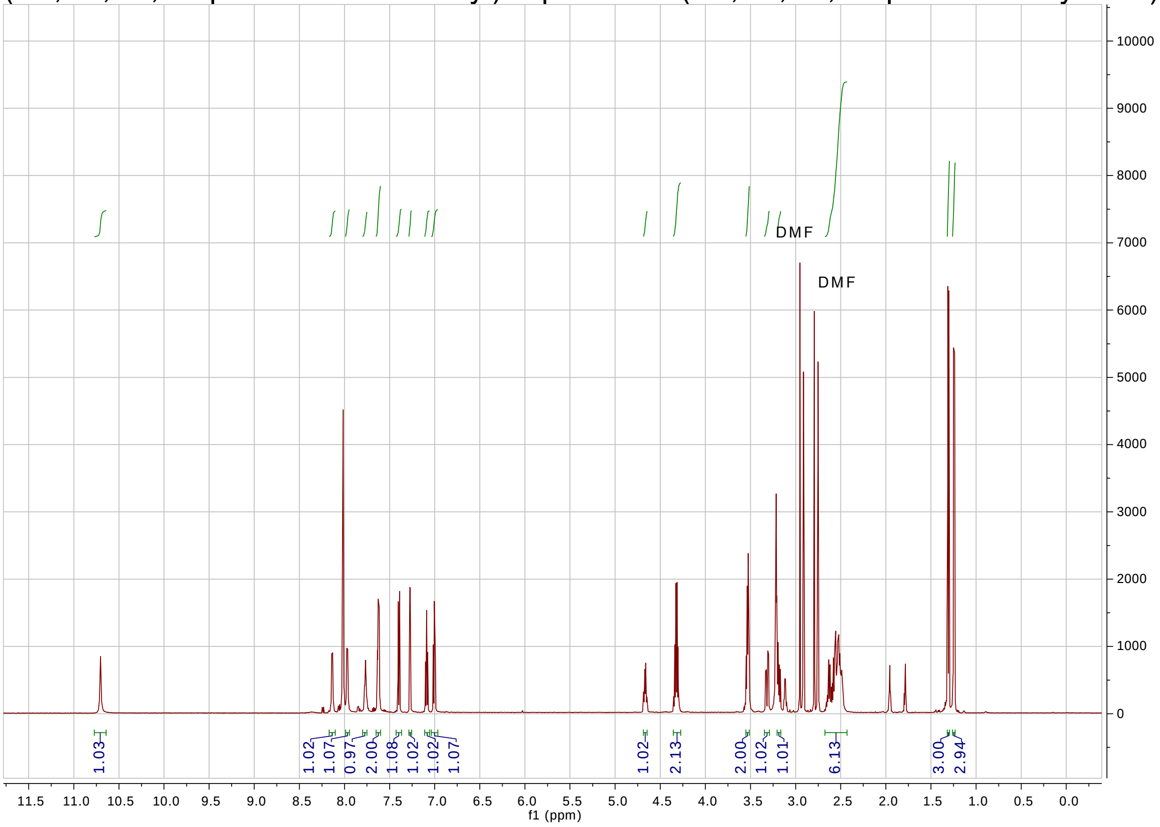

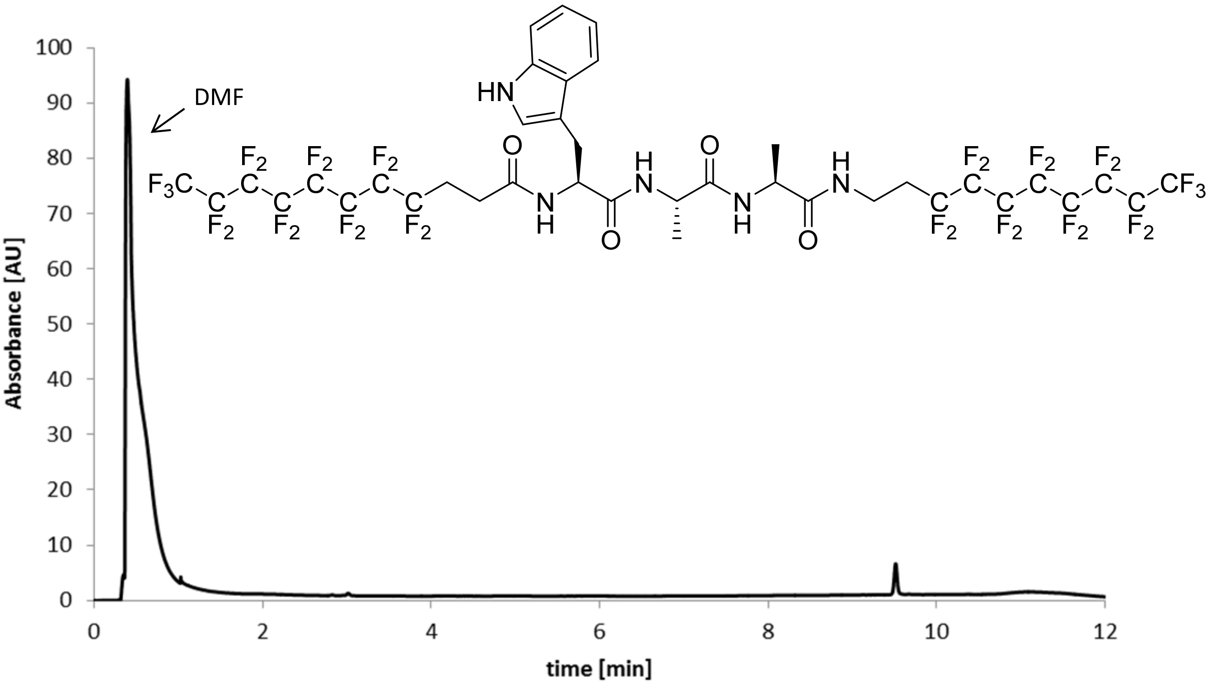

S12. (2*H*,2*H*,3*H*,3*H*-perfluoroundecanoyl)-Trp-Ala-Ala-(2*H*,2*H*,3*H*,3*H*-perfluorodecylamid) **7**. Top: ^{1}H-NMR (DMF-d_7, 600 MHz, 333 K), Bottom: UV-Vis-trace 190-500 nm of UPLC chromatogram (Method 2).

(2*H*,2*H*,3*H*,3*H*-perfluoroundecanoyl)-Ala-Ala-Trp-(2*H*,2*H*,3*H*,3*H*-perfluorodecylamid) **8**

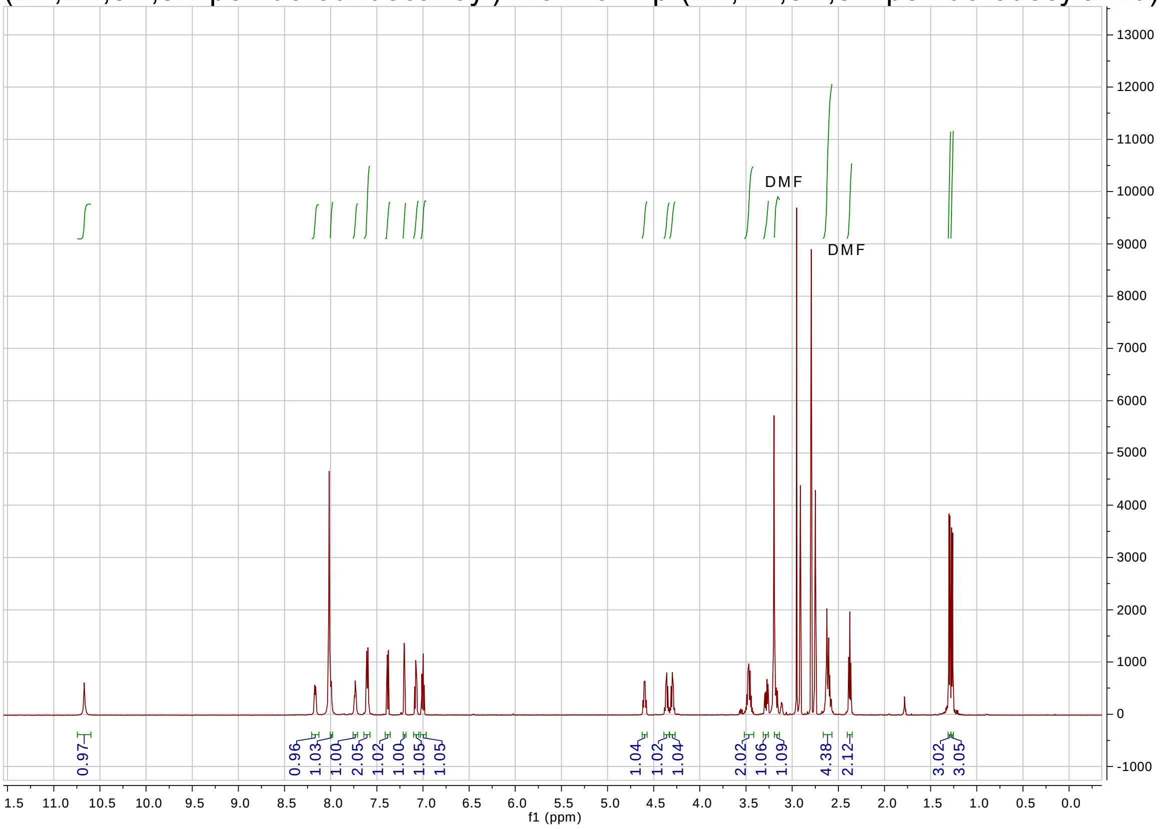

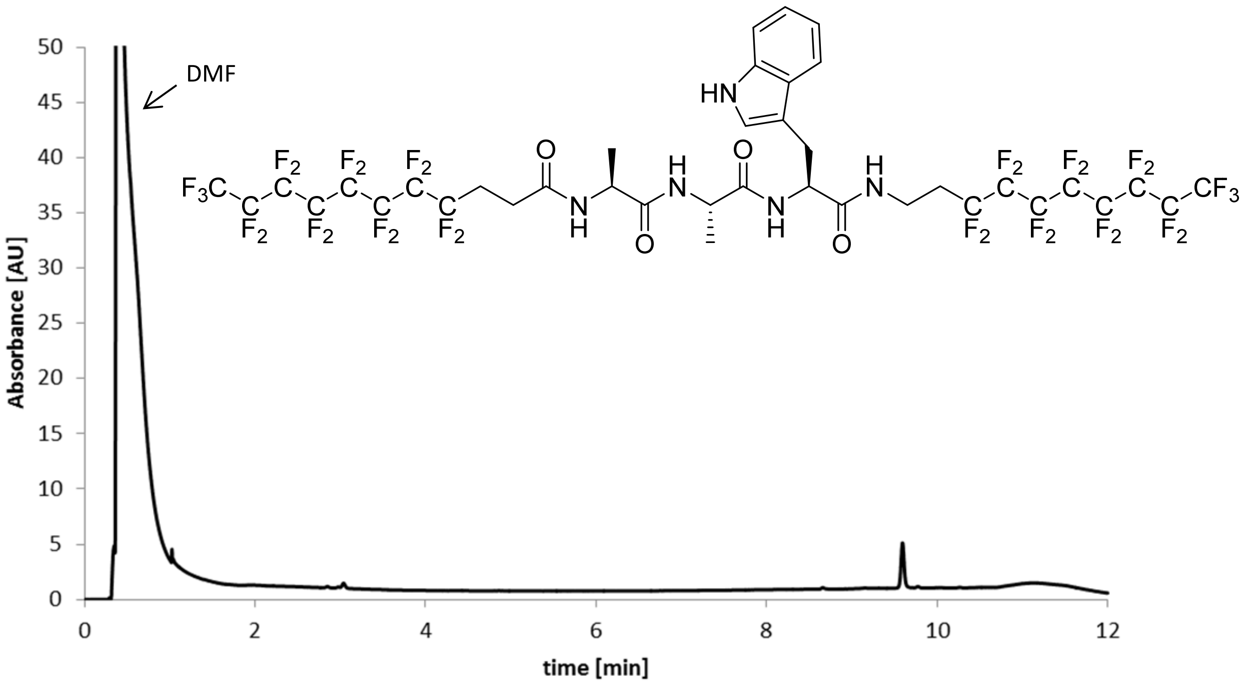

S13. (2*H*,2*H*,3*H*,3*H*-perfluoroundecanoyl) -Ala-Ala-Trp-(2*H*,2*H*,3*H*,3*H*-perfluorodecylamid) **8**. Top: ^{1}H-NMR (DMF-d_7, 600 MHz, 333 K), Bottom: UV-Vis-trace 190-500 nm (UPLC, Method 2).

NHS-ester of *2H,2H,3H,3H*-perfluorodecanoic acid

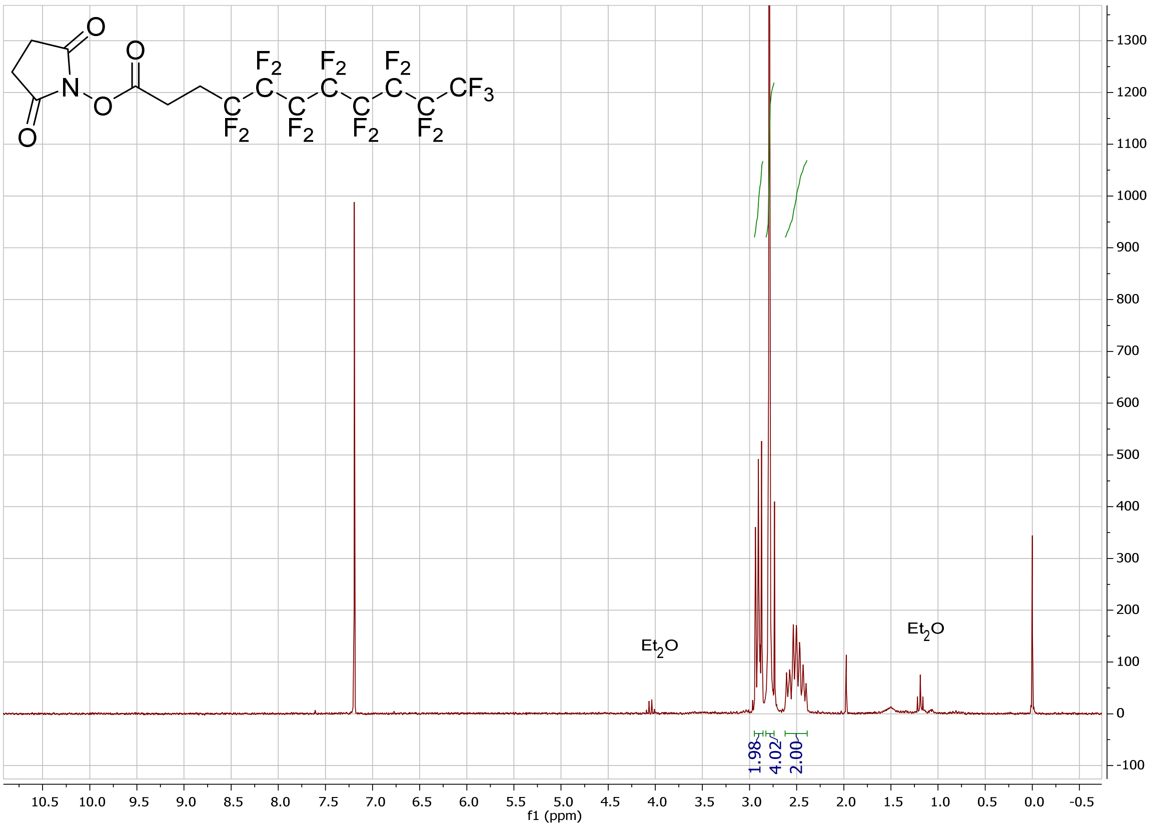

S14. *2H,2H,3H,3H*-perfluorodecanoic acid NHS ester. ^{1}H-NMR ($CDCl_3$, 500 MHz, 293 K).

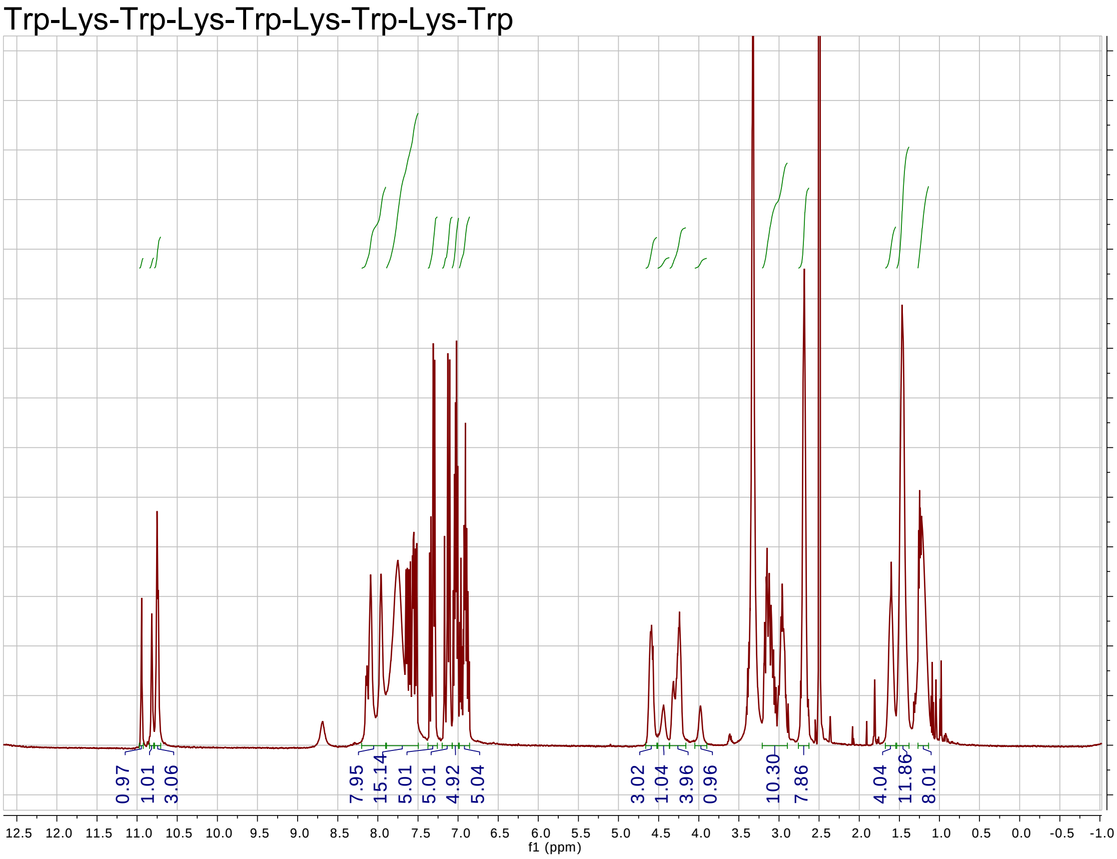

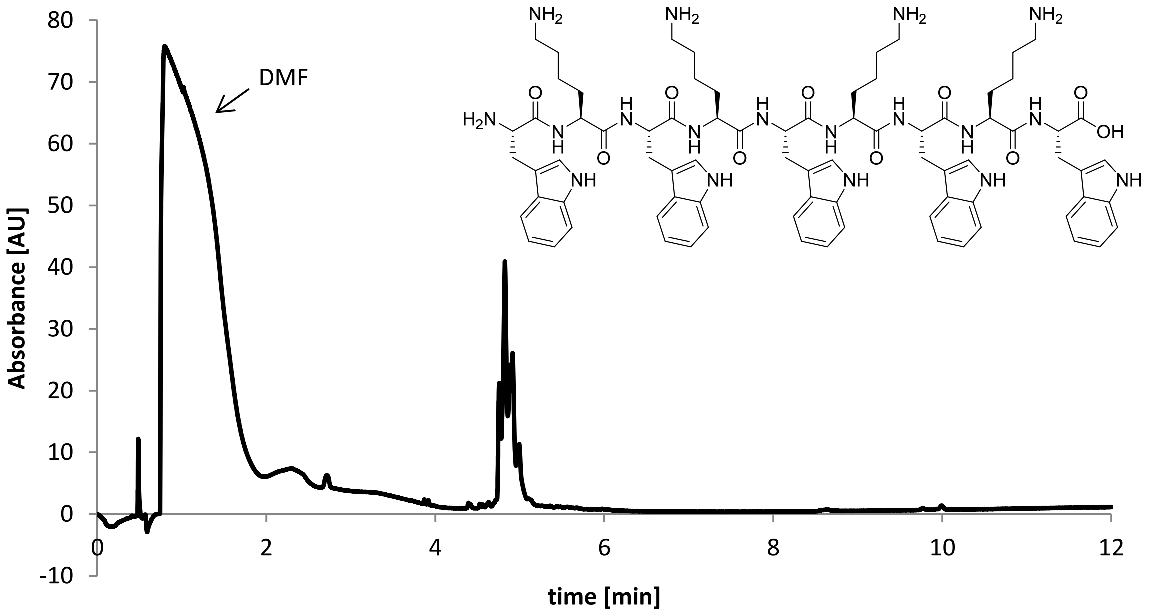

S15. Trp-Lys-Trp-Lys-Trp-Lys-Trp-Lys-Trp. Top: ^{1}H-NMR (DMSO-d$_6$, 500 MHz, 293 K), Bottom: UV-Vis-trace 190-500 nm of UPLC chromatogram (Method 1). The chromatogram shows multiple peaks with identical absorption and MS-spectra which are possibly caused by interconverting conformers.

Compound **9**

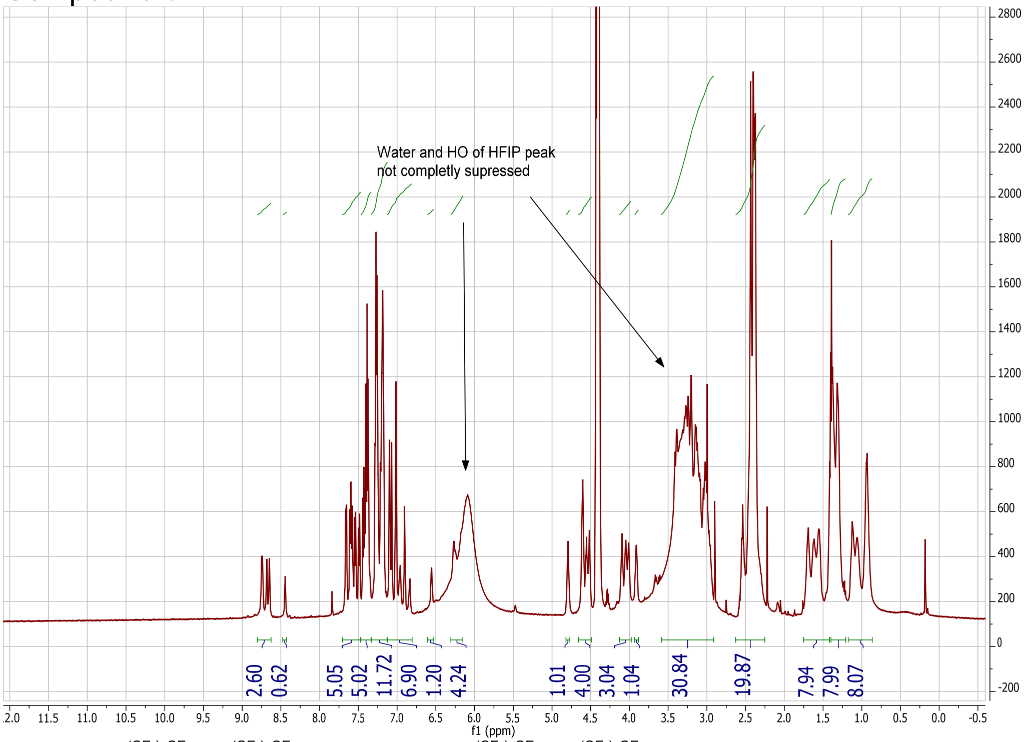

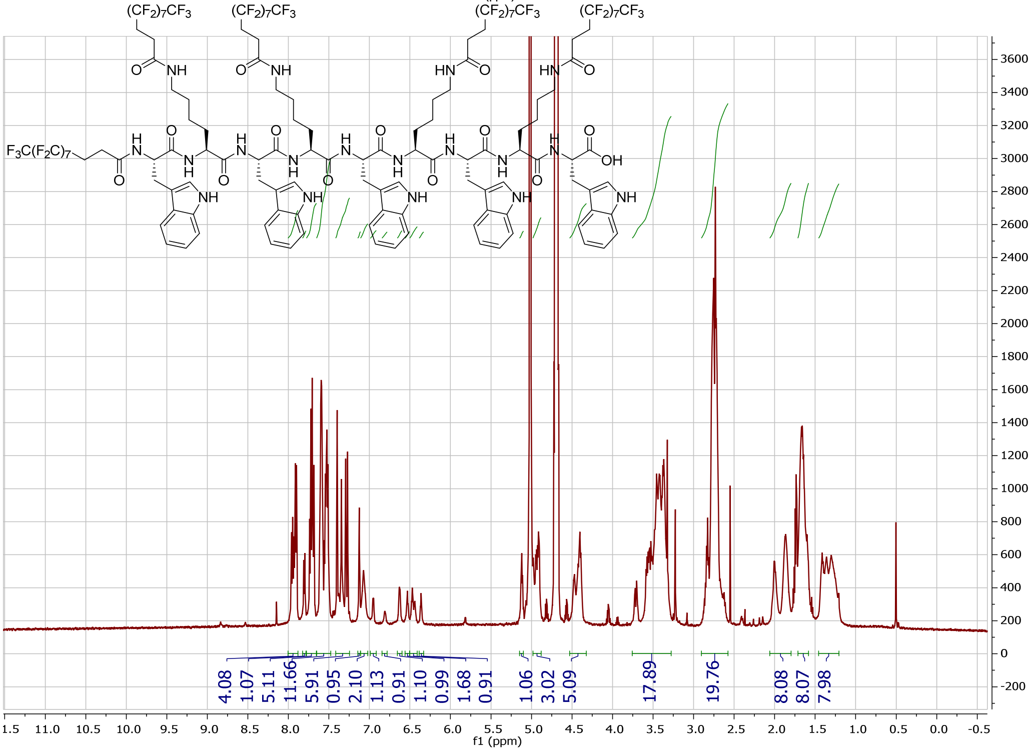

S16. Compound **9**. Top: ^{1}H-NMR (HFIP-d$_2$+ 20 mol% H2O, presat (water and OH-HFIP signal suppression, 600 MHz, 293 K), Bottom: : ^{1}H-NMR (HFIP-d$_2$, 600 MHz, 293 K).

References

[1] M. Amblard, J.-A. Fehrentz, J. Martinez, G. Subra, Methods and protocols of modern solid phase peptide synthesis. *Mol. Biotechnol.* **2006**, *33*, 239-254.

[2] H. R. Morris, D. H. Williams, R. P. Ambler, Determination of the sequences of protein-derived peptides and peptide mixtures by mass spectrometry. *Biochem. J.* **1971**, *125*, 189-201.

[3] Malik, L., Nygaard, J., Hoiberg-Nielsen, R., Arleth, L., Hoeg-Jensen, T., Jensen, K. J. Perfluoroalkyl Chains Direct Novel Self-Assembly of Insulin. *Langmuir* **2012**, *28*, 593-603.
